# Analysis of age as a factor in NASA astronaut selection and career landmarks

**DOI:** 10.1371/journal.pone.0181381

**Published:** 2017-07-27

**Authors:** Gregory T. A. Kovacs, Mark Shadden

**Affiliations:** 1 Department of Electrical Engineering, Stanford University, Stanford, California, United States of America; 2 Elite Research, LLC, Irving, Texas, United States of America; Charles P. Darby Children's Research Institute, 173 Ashley Avenue, Charleston, SC 29425, UNITED STATES

## Abstract

NASA’s periodic selection of astronauts is a highly selective process accepting applications from the general population, wherein the mechanics of selection are not made public. This research was an effort to determine if biases (specifically age) exist in the process and, if so, at which points they might manifest. Two sets of analyses were conducted. The first utilized data requested via the Freedom of Information Act (FOIA) on NASA astronaut applicants for the 2009 and 2013 selection years. Using a series of multinomial and logistic regressions, the data were analyzed to uncover whether age of the applicants linearly or nonlinearly affected their likelihood of receiving an invitation, as well as their likelihood of being selected into the astronaut program. The second used public data on age at selection and age at other career milestones for every astronaut selected from 1959 to 2013 to analyze trends in age over time using ordinary least-squares (OLS) regression and Pearson’s correlation. The results for the FOIA data revealed a nonlinear relationship between age and receiving an interview, as well as age and selection into the astronaut program, but the most striking observation was the loss of age diversity at each stage of selection. Applicants younger or older than approximately 40 years were significantly less likely to receive invitations for interviews and were significantly less likely to be selected as an astronaut. Analysis of the public-source data for all selections since the beginning of the astronaut program revealed significant age trends over time including a gradual increase in selectee age and decreased tenure at NASA after last flight, with average age at retirement steady over the entire history of the astronaut program at approximately 48 years.

## Introduction

Since 1959, NACA, and later NASA, has been selecting personnel for operational spaceflight roles. Over the history of these selections, the types and durations of missions have changed, and in selecting quality operational personnel, NASA has needed to consider variances in budgets and missions, retirements and other factors, while attempting to achieve race and gender balance. While the process is announced and conducted with fairly high visibility, the mechanics and demographics of astronaut selection are not transparent to the public nor easy to elucidate. To the best of our knowledge, this is the first effort to investigate this opaque selection process for bias.

Published objective qualifications include United States citizenship, completion of at least a Bachelor’s (engineering, science, or mathematics), management or pilot experience, and various physical requirements [1]. Age is not explicitly mentioned by NASA or the Canadian Space Agency (CSA), although the European Space Agency (ESA) states that, “the preferred age range is 27 to 37” [2].

Astronaut selections are relatively infrequent, involve large ratios of applicants to selectees, are carried out predominantly by individuals selected through the same mechanisms, and have no known external oversight. Individuals applying or selected are unlikely to criticize it, yet it is possible that inappropriate selection biases exist.

While more analysis could be done, the work reported here was restricted to the demographics of age during the selection process. Naturally, many other factors contribute to selection decisions. Considering those that might be age-dependent, it is reasonable to assume that relevant experience would increase with advancing age, yet some volatile skills (e.g., operational) may diminish, and health may deteriorate. Nonetheless, it is also reasonable to believe that equally qualified candidates significantly older than the selectees exist within the applicant pools.

It is often heard by (and from) potential applicants that they are “too old” to apply (likely leading to some degree of “self-deselection”), but it is important to ask if there is age bias in the actual selection. While there is no available evidence of mission-critical problems correlating with age alone, as will be discussed below, selectee ages over the eight selections spanning 1959 through 2013 have had an average age of approximately 34, with qualified applicant ages spanning a significantly greater age range.

In contrast to NASA astronauts, most federal operational positions have legally defined maximum applicant ages. Table 1 shows example maximum entry ages with references for many such roles. Without published maximum entry ages, employment selections must comply with the Age Discrimination in Employment Act of 1967 (ADEA, which forbids discrimination against people who are age 40 or older) [3]. Interestingly, NASA’s own criminal investigation personnel must be 37 years old or younger to apply (see Table 1) despite the lack of an upper age limit for astronauts.

**Table 1:** Maximum entry ages for federal operational positions.

Federal Employer	Max Age	Reference
United States Marine Corps	28	[4]
United States Navy	34	[5]
United States Army	35	[6]
United States Coast Guard (Aviator)	35	[7]
Dept. of Justice, Bureau of Alcohol, Tobacco, Firearms & Explosives	36	[8]
Federal Bureau of Prisons	37	[9]
United States Secret Service	37	[10]
Department of Justice, FBI	37	[11]
Forest Service Law Enforcement, Criminal Investigation	37	[12]
Department of Commerce	37	[13]
NASA, Office of Inspector General (Criminal Investigation)	37	[14]
United States Air Force	39	[15]

**Fig 1:**
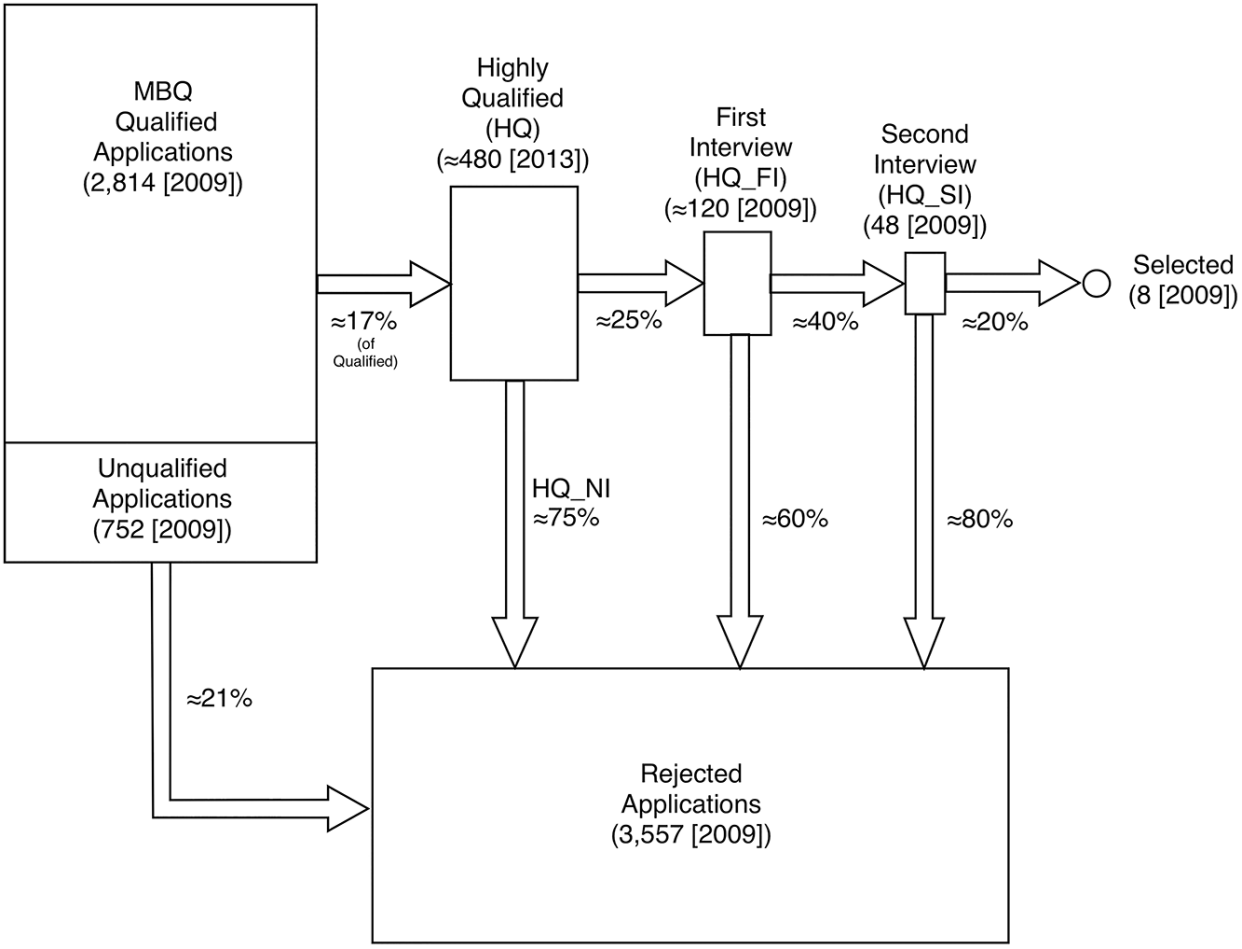
Illustration of the NASA astronaut selection process flow. Numbers of applicants at each stage are drawn from the 2009 selection data obtained via FOIA request except the HQ number which is estimated from the 2013 cohort wherein it was 483 persons. Approximate numbers of applicants moving between each sub-group are stated as percentages and include medical disqualifications (occurring during the interview process), which were not identified in the data provided by NASA. The geometric areas of the shapes representing each group are approximately to scale.

While the mechanics of the process are not made public by NASA, data from two FOIA requests made as part of this research were used to create an overview of the selection procedure, illustrated in Fig 1.

When a selection cycle is announced, applications are accepted by NASA over several months (11 months for 2009, 2.5 months for 2013, and 2 months for the 2016 selection). In prior selections, applications were processed using paper through the mail, but for the 2009 and subsequent selections, an online application through USAJOBS.gov was used [16]. Referring to Fig 1, based primarily on the 2009 data which includes ages for all records, approximately 21% of the applications received were not valid (which here is defined as not meeting the minimum Bachelor’s degree requirement published by NASA). Those meeting this minimum requirement are referred to herein as “Meets Basic Qualifications” (MBQ). From the total applicant pool of 3,566 persons, approximately 17% were deemed “Highly Qualified” (HQ) by a process which for those newly applying can only be based on information they submitted (and thus should be objective). As discussed below, however, a person rated HQ can be demoted from HQ status on a subsequent application following an interview and possibly without one.

HQ status is necessary for progression, since it is a required but not sufficient condition for the interview. Invitation for interview is the only path toward selection into the astronaut program, and thus analyses of demographics of the HQ group and the interview group were considered likely to illuminate any potential biases. Approximately 120 HQ applicants are invited for a first round of interviews (approximately 2.5 days of interviews and medical testing), followed by a smaller group (48 in 2009) invited for a second round of interviews (week-long, with more extensive medical and psychological testing) from which the selectees are drawn.

Some important factors are not captured in the flow diagram in Fig 1. First, many applicants have applied multiple times prior to an interview being granted, and it is not clear by what mechanisms their status might or might not advance (but by definition they must retain HQ status). Secondly, many non-selected interviewees are “recycled” in subsequent selection(s) and some of these individuals are then selected. For example, in the 2013 selection 20 individuals (16.7% of the interviewee pool) had been interviewed in a previous selection cycle (2009 or earlier). It is not clear by what mechanism they are advanced through the process to another interview despite a new and sometimes much larger pool of applicants in the next cycle. “Recycling” of interviewees has a marked effect on blocking those who have not yet had an interview from that necessary (though not sufficient) step toward selection. Interestingly, 3 of the 8 selectees in 2013 had been finalists in the 2009 selection and 25 who were interviewed in 2009 were demoted from HQ status in 2013 when re-applying.

To test for possible age biases at each stage of selection, data from the two FOIA requests (source data files S1 and S2 Files) were analyzed to compare the age demographics of the pools at each available selection stage. In addition, publicly available data from astronaut biographies [17] (and other sources on the internet, as noted in the source data file, S3 File) were compiled and analyzed to provide insight into trends in age demographics of selection and other important career milestones in the astronaut corps since its inception.

## Materials and methods

Throughout this work, database functions and data management were carried out using Microsoft Excel 2011 (Microsoft Corporation, Redmond, WA) with statistical analysis using Stata 14.1 (StataCorp LP, College Station, TX). Data visualization was done using R 3.2 (R Core Team, R Foundation, Vienna, Austria), and the RStudio package ggplot2 1.0.1 (RStudio, Boston, MA) [18].

### 2009 and 2013 selection data description and preparation

Data for the 2009 and 2013 selections were obtained via independent FOIA requests. Due to differences in the way requests were made and their interpretations by NASA, incomplete and sometimes erroneous data records lead to differences in the applicability of the datasets.

Although the 2009 data do not contain HQ or overall interview status, each record contains full birthdate, gender, race, academic degrees, NASA non-astronaut employment history, and military status. A subset of the 2009 data, identifying 48 applicants who received a second interview, provided insight into the last interview phase.

The 2013 data includes gender, race, academic degrees, HQ and interview status, whether or not an applicant had received a previous interview, but does not include military service and NASA experience information. Importantly, a significant portion of applicant records in the 2013 data do not have birthdate reported.

As stated in the FOIA response from NASA, due to the use of the USAJOBS on-line application process for 2013, age data was apparently not collected for all applicants (notably, however, USAJOBS was also used in 2009 and age data *was* collected for all applicants). In the 2013 data, age information was provided for almost all HQ, interviewee and selectee personnel, as well as gender, race, and degree(s). Due to these discrepancies, the data for 2009 and 2013 were analyzed separately. However, these differences allowed for analyses answering distinct research questions about the astronaut selection process.

For consistency, all ages were calculated relative to the date of each group’s official selection announcement date. For 2009, all 3,566 records had full birthdates. For the 2013 data, 893 out of 6,113 records had birthdate information. Records without valid ages were then removed from the analysis pools. Infant and child age outliers were removed: 19 records for 2009 and 2 for 2013 had ages of 0.5 through 12.5 years, leaving 3,547 and 891 records, respectively. Those lacking the minimum published Bachelor’s degree were then removed; 752 were removed from the 2009 data and 13 from the 2013 data. It was noted that for 2009, 21% of the applications were lacking the minimum degree and for 2013, 1,560 of the total records, or 26%, were also lacking it. Cross-checks resulted in the realization that one record, for K. Lindgren (DOB 1/23/73) had been mis-coded as a 2008 DOB by NASA, so this record was corrected and added back to the dataset. The final useable record counts were 2,796 for 2009 and 878 for 2013.

Since the reason for such a large proportion of missing age values in the 2013 data was not clear, the analyses were carried out under the assumption that the missing age values are missing at random and the absence of this data is not associated with any predictors of interview or selection status.

Aside from the main independent variable, age, other covariates of interest included in the analysis were: education level, NASA experience, military service, and gender. Education level and gender are available for both the 2009 and 2013 data. Education level was measured as the number of years of postsecondary education completed by the applicant with Bachelors equal to 4 years, Masters equal to 2 years, and Ph.D. or M.D. equal to 4 years. Gender is a dichotomous variable with female as the reference category. As noted above, NASA experience and military service were only made available for the 2009 data. Both are dichotomous variables measuring whether the applicant ever had NASA non-astronaut employment or military service, respectively. Race was deliberately not considered in these analyses.

The most significant difference in analyses between the datasets was the operationalization of the dependent variables. The outcome of interest is how far an applicant proceeds through the selection process. As outlined in Fig 1, there are five stages at the core of the selection process. In order for an applicant to be in the sample for this analysis, they must at least reach MBQ status. Once the pool of MBQ applicants has been identified by NASA, an undisclosed procedure is carried out to determine which applicants are HQ (with new applicants’ HQ status being determined without consideration of prior interview). Applicants who are not listed as HQ cannot advance to the first interview. Next, another set of undisclosed criteria are used to determine which of the HQ applicants are invited to the first interview, thus reducing the remaining applicant pool further. Among those invited for the first interview, another, smaller group advances to the penultimate step in the process, a second interview. The final stage is selection into the NASA astronaut program.

Given the description of the outcome of interest above, an appropriate measurement of the outcome variable would be an ordinal measure with five categories: MBQ not interviewed (MBQ_NI), HQ not interviewed (HQ_NI), HQ granted first interview (HQ_FI), HQ granted second interview (HQ_SI), and selectee (S). However, as noted above, the data only contain limited information for each of the five categories, and HQ_FI was not explicitly available.

The 2009 data only identify two categories precisely: selectee (S) and HQ invited to second interview (HQ_SI), and other categories imprecisely, with no distinction between MBQ and HQ. Due to this lack of distinction between some categories, two dependent variables for the 2009 data were operationalized. One dependent variable was measured as a categorical/ordinal variable with the categories no final interview (*N* = 2,748), final interview not selected (*N* = 39), and selected (*N* = 9). However, due to the relatively low frequency of selectees (0.32%), an additional dichotomous dependent variable was operationalized as no final interview (*N* = 2,748) and final interview (*N* = 48). Although the final interviewees (1.72%) still appear rather infrequently relative to those who did not receive a final interview (98.28%), this operationalization slightly improves the balance in frequencies, while also allowing investigation into a distinct component of the selection process (how age impacts the step from no interview to final interview).

The 2013 data also fall short of clearly identifying each of the five categories in the astronaut selection process, although the difference between the categorizations provided for 2013 relative to the categories provided for 2009 again allow for investigation into distinct questions about the role of age in the selection process, thus incrementally providing a fuller picture of the process. Records with age information from 2013 precisely identify categories for MBQ not interviewed (MBQ_NI) (*N* = 431; 49.09%), HQ not interviewed (HQ_NI) (*N* = 332; 37.81%), HQ rejected after *at least* the first interview (HQ_RAAFI) (*N* = 107; 12.19%), and selectee (*N* = 8; 0.91%). This operationalization was used for analysis, although again due to the low relative frequency of selectees and to expand understanding of the selection process, a second operationalization was used: HQ_NI (*N* = 332; 74.27%) and HQ interviewee (HQ_I) (*N* = 115; 25.73%). Although not ideal, the results of analyses with these four operationalizations of the selection process can be taken together to provide a more holistic view of the role played by age in astronaut selection.

### Historical data (1959–2013) description and preparation

In order to explore whether or not the average age of astronauts has changed systematically over time, data were acquired on the ages of astronauts at time of selection at other key milestones over the entire history of NASA’s human space program. Data were collected manually from the NASA website [17] (and cross referenced with Wikipedia [19] and Space Facts [20] websites, as noted in the source data file, S3 File) on the ages at selection of all 327 NASA astronauts from the first group of selectees in 1959 to the most recent group in 2013. A manual validation check was carried out for this data, and any discrepancies between the three sources were followed up with research for additional information until a confirmation could be obtained. The acquired information is current to January 31, 2017.

Although ages for the selection process (2009 and 2013) were calculated to the day, the month and day of birth were not available for some astronauts in earlier cohorts. Where only the year and month were available, day was estimated as the middle of the relevant month. Where only year was provided, the approximate midpoint date of that year was used. The age variable for the full NASA astronaut selectee pool was measured in decimal years. Month and day were generally not provided for retirements, and the same approximations were employed as needed.

Data collected included each person’s active status (currently active, not active), gender, advanced degrees obtained (M.S., Ph.D., M.D.), military experience, previous NASA experience, and mission roles (i.e., pilot, payload specialist, mission specialist, etc.). Key dates of interest were also collected including, selection date (official selection announcement dates could not be obtained for 22 individuals), date of birth, date of death (if applicable), first flight date, last flight date, and retirement date (if applicable). For dates with only a year or month or only year available rather than an exact date, the middle of the year (or month) was tabulated. Since retirement data is not consistently available from public sources, NASA was contacted by telephone and responded on 12/5/16 that they do not maintain accessible retirement data for public access. Many astronauts are still employed at NASA today and several died while employed by NASA (i.e., neither group retired), some before any space mission, with suitable notations also tabulated. Only relevant astronauts were included in subsequent analyses. From this information, calculations were made to compute each astronaut’s age at selection, first flight, last flight and retirement versus selection year; and elapsed time from selection to first flight, first to last flight, first flight to retirement, selection to last flight, last flight to retirement, and selection to retirement.

### 2009 and 2013 selection data analysis methods

Preliminary analyses were performed on the 2009 data using an independent samples t-test and one-way ANOVA to assess whether there were differences in the average age of NASA astronaut applicants across the categories of selection stage described in the previous section. Again, it is important to note that the 2009 data provided through the FOIA request do not distinguish between MBQ and HQ applicants who were not selected for the final interview, therefore, inferences can only be drawn about the applicant pool as a whole.

Although HQ and MBQ were not distinguished in the 2009 data, HQ status is necessary but not sufficient to receive an invitation for an interview. Those applicants who received an invitation are inherently HQ, but the MBQ/HQ status of those applicants who did not receive an invitation cannot be discerned from the data, therefore, they were referred to as MBQ (an MBQ/HQ comparison is presented in the following section using the 2013 data). Applicants who did not meet the basic requirements (i.e., no post-secondary education) were excluded from the analysis.

ANOVA and t-test analyses are, however, limited to comparing only the means of different groups. Predicting the probability of selection across age is a more valid test of the hypothesis. A series of logistic regressions were used to predict the likelihood that an applicant was chosen for the final interview across all ages of the qualified applicants.

Since the data under investigation include both dichotomous and categorical dependent variables, logistic regression and multinomial logistic regression models were used for the majority of primary analyses. A logistic regression uses one or more independent variables to predict the likelihood of an event when the event is operationalized as a dichotomous variable. The formulation of a logistic regression is displayed below, where *lnL* represents the log-likelihood of the event occurring (*Y = 1*) given the estimation of the intercept’s, *β*_*0*_, and all other independent variables’, *β*_*1…k*_, impact on the log-likelihood. These log-likelihood estimates are then converted to interpretable odds ratios by exponentiating their estimated values.

$$lnL\left(Y=1\right)=\beta_{0}+\beta_{1}X_{1}+\beta_{2}X_{2}+\cdots \;\beta_{k}X_{k}$$

Multinomial logistic regression is an extension of logistic regression formulated to account for categorical dependent variables with more than two categories. After deciding on a reference category, typically *Y = 0*, a series of logistic regressions are estimated in a unified framework in order to determine the impact each of the independent variables have on the likelihood of observing each outcome or type of event, *Y = 1…k*, relative to the likelihood of observing the outcome *Y = 0*.

$$\begin{array}{l} lnL\left(Y=1\right)=\beta_{0}+\beta_{1}X_{1}+\beta_{2}X_{2}+\cdots \;\beta_{k}X_{k}\\ lnL\left(Y=2\right)=\beta_{0}+\beta_{1}X_{1}+\beta_{2}X_{2}+\cdots \;\beta_{k}X_{k}\\ \cdots \\ lnL\left(Y=k\right)=\beta_{0}+\beta_{1}X_{1}+\beta_{2}X_{2}+\cdots \;\beta_{k}X_{k} \end{array}$$

Initially, a baseline model was specified using only age as a predictor of selection for the final interview, then the covariates (education, NASA experience, military service, and gender) were included to more fully specify the model.

This two-step process was performed again using logistic regression with only a random sample of 400 applicants who were not selected for the final interview and all 48 applicants who were selected for the final interview. King [21], and King and Zeng [22] demonstrated that logistic regression models performed on a dependent variable with extreme imbalance in frequencies (e.g., 95% zeros and 5% ones) can lead to various issues with model validity; therefore, their suggested procedure was performed as a sensitivity analysis since the imbalance in the dependent variable was approximately 98% zeros and 2% ones. Additionally, Firth exact logistic regression was performed to address the dependent variable imbalance.

Nonlinear relationships may, however, exist between parameters (some scholars have even suggested that there may be fewer linear relationships in the universe than nonlinear ones [23, 24]). To this end a quadratic relationship between age and final interview selection was explored by including a squared term for age into the logistic regression [25].

Multinomial logistic regressions were performed to investigate if the nonlinear relationship also exists between age and the additional dependent variable in the 2009 data, final selection status. Final interview status contains three categories: not selected for final interview (both MBQ and HQ combined); selected for final interview (by definition, HQ), and selected into the astronaut program. Because the dependent variable is categorical with more than two categories, it is necessary to use multinomial logistic regression, a maximum likelihood estimation extension of logistic regression, in lieu of logistic regression. With logistic regression, there are only two categories, thus the lower category is assumed as the reference category by statistical software, multinomial models must be explicitly informed of the chosen reference category. All qualified applicants not selected for the final interview in 2009 were set as the reference category for comparison with applicants who were selected for the final interview but were ultimately rejected, and applicants who were selected for the astronaut program.

### Historical data (1959–2013) analysis methods

Relative to the 2009 and 2013 FOIA data sets, analyses of which were complicated by differing information content, the manually collated historical data over all selections was consistent, thus only requiring the use of basic and well-known statistical methods.

Descriptive statistics (mean, standard deviation, min/max) were calculated for historical selection data for age at selection, first flight, and last flight, and retirement; as well as time from selection to first flight, first to last flight, first flight to retirement, selection to last flight, last flight to retirement, and selection to retirement.

The data were also analyzed using pairwise Pearson’s correlations of age at selection, first flight, last flight and retirement versus selection year, and time from selection to first flight, first to last flight, first flight to retirement, selection to last flight, last flight to retirement, and selection to retirement.

Ordinary least-squares (OLS) regression was also carried out over age at selection, first flight, last flight and retirement, with variables of selection year, gender, military experience, previous NASA employment, doctoral degree (Ph.D., M.D.), role (pilot, mission specialist, payload specialist), and eventual management astronaut role.

## Results and discussion

### 2009 NASA astronaut selection process

As described above, an independent samples t-test was used to determine if there was a statistically significant difference between the average age of MBQ applicants who were not invited to the final interview and applicants who were invited to the final interview. The results indicate that there is no significant difference in the average age between applicants who were invited for the final interview and applicants who were not invited (see Fig A in S1 Appendix). Both groups had an average age of approximately 38 years old. A one-way ANOVA was also performed to compare the average ages across the three groups of applicants who were not invited for the final interview, applicants who were invited to the final interview, but were not ultimately selected for the astronaut program, and applicants who were selected for the astronaut program. Again, no evidence was found to indicate any significant difference in average age across groups (see Fig B in S1 Appendix).

A series of logistic regressions were used to predict the likelihood that an applicant was chosen for the final interview across all ages of the qualified applicants. As noted above, a baseline model was specified using only age as a predictor of selection for the final interview, followed by a full model with the covariates (education, NASA experience, military service, and gender) included.

Across all model specifications, age had no effect on the probability of being chosen for a final interview among all applicants, whether MBQ or HQ (see Table A in S2 Appendix). Firth exact logistic regression (to address the dependent variable imbalance) produced results that remained substantively unchanged.

One result to note is the rather large effect of military experience on likelihood of being chosen for a final interview. Applicants with military service experience were approximately *ten times more likely* to be chosen for a final interview than were applicants without military service experience, even after controlling for age.

Another series of logistic regressions were performed with the inclusion of a quadratic term for age (age^2^). The results of the quadratic models, in Table 2, indicate that there is indeed a nonlinear relationship between age and the odds of being selected for the final interview.

**Table 2:** Logistic regression predicting selection for final interview with quadratic age (2009 data).

	Full Sample		Random Sample	
	Base Model	Full Model	Base Model	Full Model
Age	3.14***	2.22**	2.91***	2.13**
	(1.084)	(0.757)	(0.990)	(0.711)
Age Squared	0.99***	0.99**	0.99***	0.99**
	(0.004)	(0.004)	(0.004)	(0.004)
Education Level		1.14***		1.17***
		(0.052)		(0.062)
NASA Experience		2.19*		2.72*
		(1.025)		(1.410)
Military Service		8.28***		9.73***
		(2.834)		(3.901)
Male		0.69		0.85
		(0.273)		(0.360)
Intercept	< 0.01***	< 0.01***	< 0.01***	< 0.01***
	(< 0.001)	(< 0.001)	(< 0.001)	(< 0.001)
Observations	2,796	2,796	448	448

Base Models explore only the effects of age on selection for final interview. Full Models incorporate potentially confounding covariates. Odds ratios are reported. Standard errors are in parentheses.

*** p<0.01,

** p<0.05,

* p<0.1

**Fig 2:**
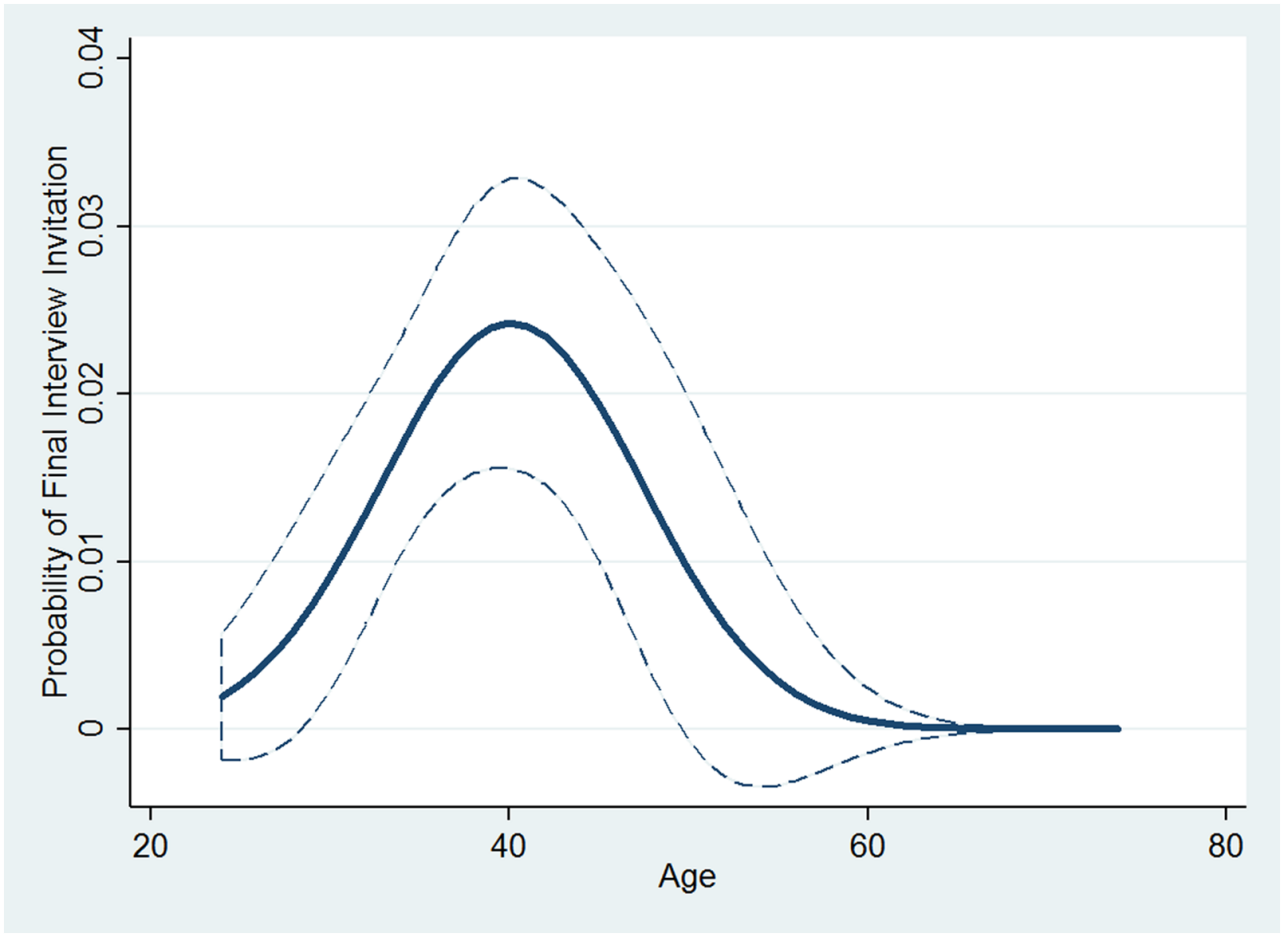
Predicted probabilities of final interview invitation across quadratic age (2009 data). All other covariates were held at their means. Dashed lines indicate 95% confidence intervals.

Although the results from Table 2 indicate a statistically significant, nonlinear relationship between age and likelihood of final interview invitation, the exact nature of the relationship is more easily discerned using visualizations. Fig 2 displays the predicted probabilities of being selected for the final interview on the y-axis against the full age range of qualified applicants on the x-axis. As shown in the graph, the likelihood of being chosen for the final interview is quite low for applicants younger than 30 and older than 50; however, as an applicant’s age increases from 30 to 40, or decreases from 50 to 40, their likelihood of being chosen for the final interview increases considerably. This provides evidence that there is not only an empirical age bias against older qualified applicants in the final interview selection, but there is also evidence of bias against younger applicants.

Although younger applicants as well as older applicants are less likely to be selected for the interview than are middle-aged applicants, the causal dynamics are not likely to be the same. For example, applicants younger than 30 may be perceived as too inexperienced for astronaut selection, whereas concerns about physical condition or reduced potential operational career span may be raised for applicants above the age of 50.

The results of multinomial logistic regressions on the 2009 data indicate that there was no linear relationship between age and final selection status (see Table B in S2 Appendix); however, inclusion of a quadratic term for age revealed evidence that the nonlinear relationship also extended to final selection status (see Table 3). The results echo the findings of the previous logistic regressions, indicating that age has a nonlinear relationship with selection for the final interview among all applicants, but there is no nonlinear relationship between age and final selection into the astronaut program. However, it is important to note that the extremely small sample size for those selected into the program (N = 9) may be a factor influencing the null findings.

**Table 3:** Multinomial logistic regression predicting final selection status with quadratic term (2009).

	Full Sample		Random Sample	
	Base Model	Full Model	Base Model	Full Model
**Rejected at Final Interview**				
Age	3.00***	2.19**	2.81***	2.14**
	(1.110)	(0.803)	(1.015)	(0.766)
Age Squared	0.99***	0.99**	0.99***	0.99**
	(0.005)	(0.005)	(0.005)	(0.004)
Education Level		1.13**		1.16***
		(0.056)		(0.066)
NASA Experience		2.69**		3.26**
		(1.288)		(1.713)
Military Service		7.20***		8.34***
		(2.741)		(3.624)
Male		0.93		1.11
		(0.430)		(0.544)
Intercept	< 0.01***	< 0.01***	< 0.01***	< 0.01***
	(< 0.001)	(< 0.001)	(< 0.001)	(< 0.001)
**Selected into Astronaut Program**				
Age	5.08	2.86	4.47	2.23
	(5.220)	(2.825)	(4.515)	(2.012)
Age^2^	0.98	0.99	0.98	0.99
	(0.013)	(0.013)	(0.0132)	(0.012)
Education Level		1.21*		1.27**
		(0.131)		(0.148)
NASA Experience		< 0.01		< 0.01
		(0.005)		(0.002)
Military Service		15.26***		20.31***
		(11.802)		(18.041)
Male		0.24*		0.31
		(0.189)		(0.251)
Intercept	< 0.01*	< 0.01	< 0.01*	< 0.01
	(< 0.001)	(< 0.001)	(< 0.001)	(< 0.001)
Observations	2,796	2,796	448	448

Base Models explore only the effects of age on selection for final selection status. Full Models incorporate potentially confounding covariates. MBQ and HQ but not invited for the final interview is the reference category. Relative risk ratios are reported. Standard errors are in parentheses.

*** p<0.01,

** p<0.05,

* p<0.1

**Fig 3:**
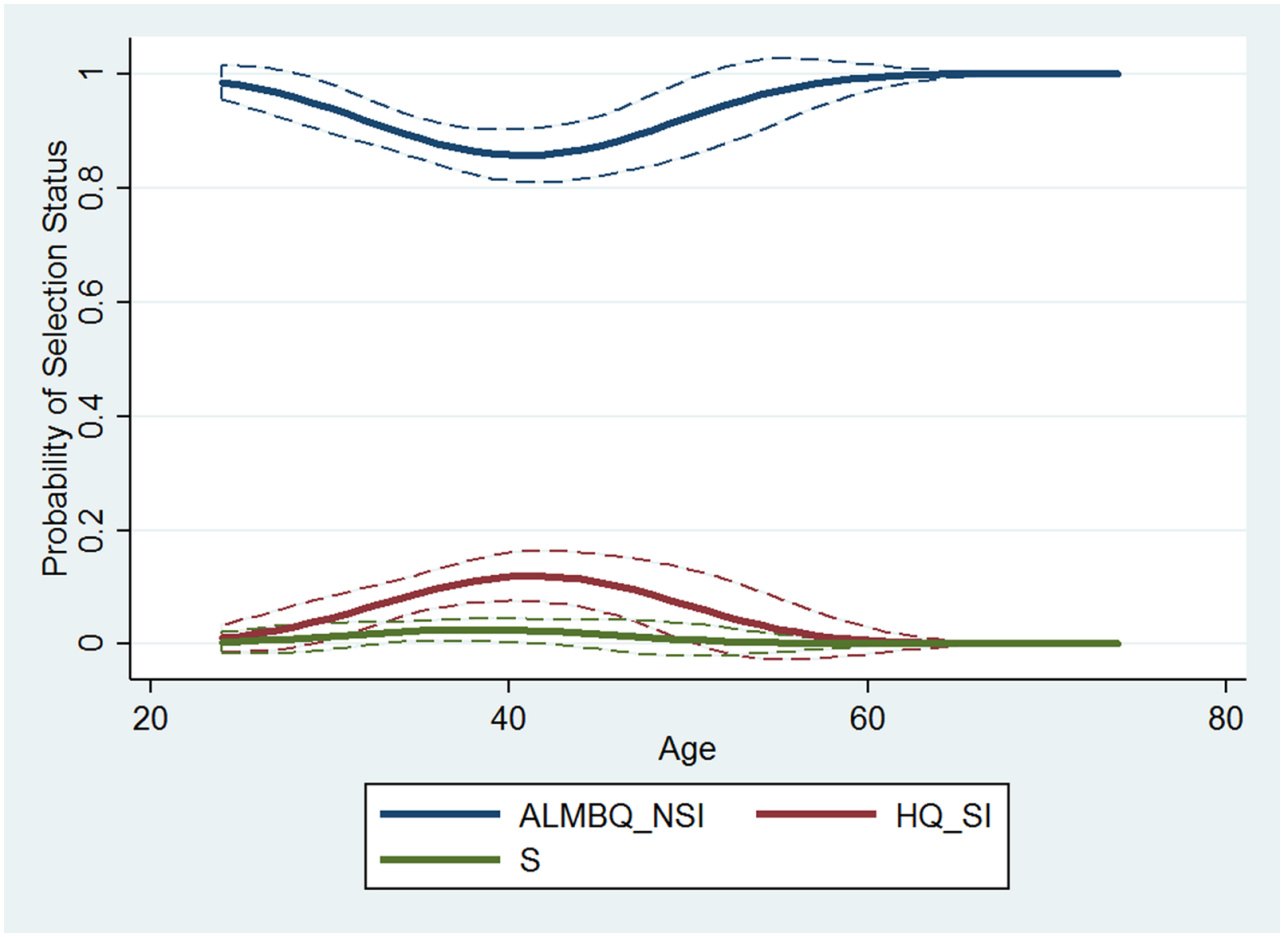
Predicted probabilities of final selection status across quadratic age (2009). Dashed lines indicate 95% confidence intervals. (Variables are defined as follows: S = Astronaut Program Selectee, HQ_SI = Highly Qualified and Granted a Second Interview, and ALMBQ_NSI = At Least Met Basic Qualifications but Not Granted a Second Interview.)

A predicted probability plot was prepared to more clearly communicate the nuanced relationship between age and final selection status (see Fig 3). Although the probability of selection into the astronaut program among all applicants in 2009 remains relatively flat across all ages (i.e., the likelihood of selection remains constant with age), the probability of being selected for the final interview among all applicants in 2009 peaks near the age of 40 and gradually declines as applicants are older or younger.

### 2013 NASA astronaut selection process

Before exploring nonlinear relationships in the 2013 data, linear relationships were investigated using the same procedure employed with the 2009 data. An independent samples t-test revealed a statistically significant, but very small difference in age between applicants who were invited for any interview and those who were not, specifically among the subsample of applicants who were classified as HQ. HQ applicants who did not receive any interview invitations were slightly older (1.2 years), on average, than HQ applicants who were invited for at least one interview (see Fig C in S1 Appendix). Although this is a statistically significant difference in average age across the groups (p-value = 0.036), it is quite small in magnitude. The recently published statement on the misuse of p-values by the American Statistical Association (ASA) highlights the importance considering effect size, rather than solely relying on p-values for scientific discovery [26]. Due to this extremely low effect size between age and interview selection (Cohen’s d = 0.23), this finding can be dismissed as negligible.

Additionally, the 2013 data contain a 4-level categorical variable indicating whether the applicant was MBQ, HQ_NI, HQ and rejected after an interview (HQ_IR), or S. A one-way ANOVA was used to determine whether there were significant differences in average ages across these categories (see Fig D in S1 Appendix). The results of the analysis indicate that there are significant differences in average ages across categories. A Tukey post hoc test revealed that there are only 2 significant differences across groups. On average MBQ-only applicants are significantly older than HQ applicants who were not interviewed by approximately 3 years. Also, on average, MBQ-only applicants were approximately 4 years older than HQ applicants who were interviewed, but ultimately rejected. No other pair of categories had statistically significant differences in average ages. Although the average age of applicants selected for the astronaut program is even younger than HQ applicants who were rejected, the standard error around the estimate of the average age for applicants who were selected is much larger due to a relatively low sample size of 8, which ultimately leads to a statistically insignificant difference between selected applicants and applicants in the other categories. (Predicted probabilities of final selection status across linear age are shown in Fig E in S1 Appendix.)

Although a small, but significant direct difference in average ages was revealed via linear analysis, it was considered that there could still be nonlinear relationships between age and interview selection of final selection status, and the effects may be even larger. Due to the differences in variable measurement between the 2009 and 2013 data described above, the inferences drawn in analyses of the 2013 data were more nuanced by qualification level. Additionally, neither NASA experience nor military service experience information was available in the 2013 data, therefore, those variables could not be included as covariates in these models. However, education level and gender were used as covariates in the primary multivariate models for 2013 applicants.

**Fig 4:**
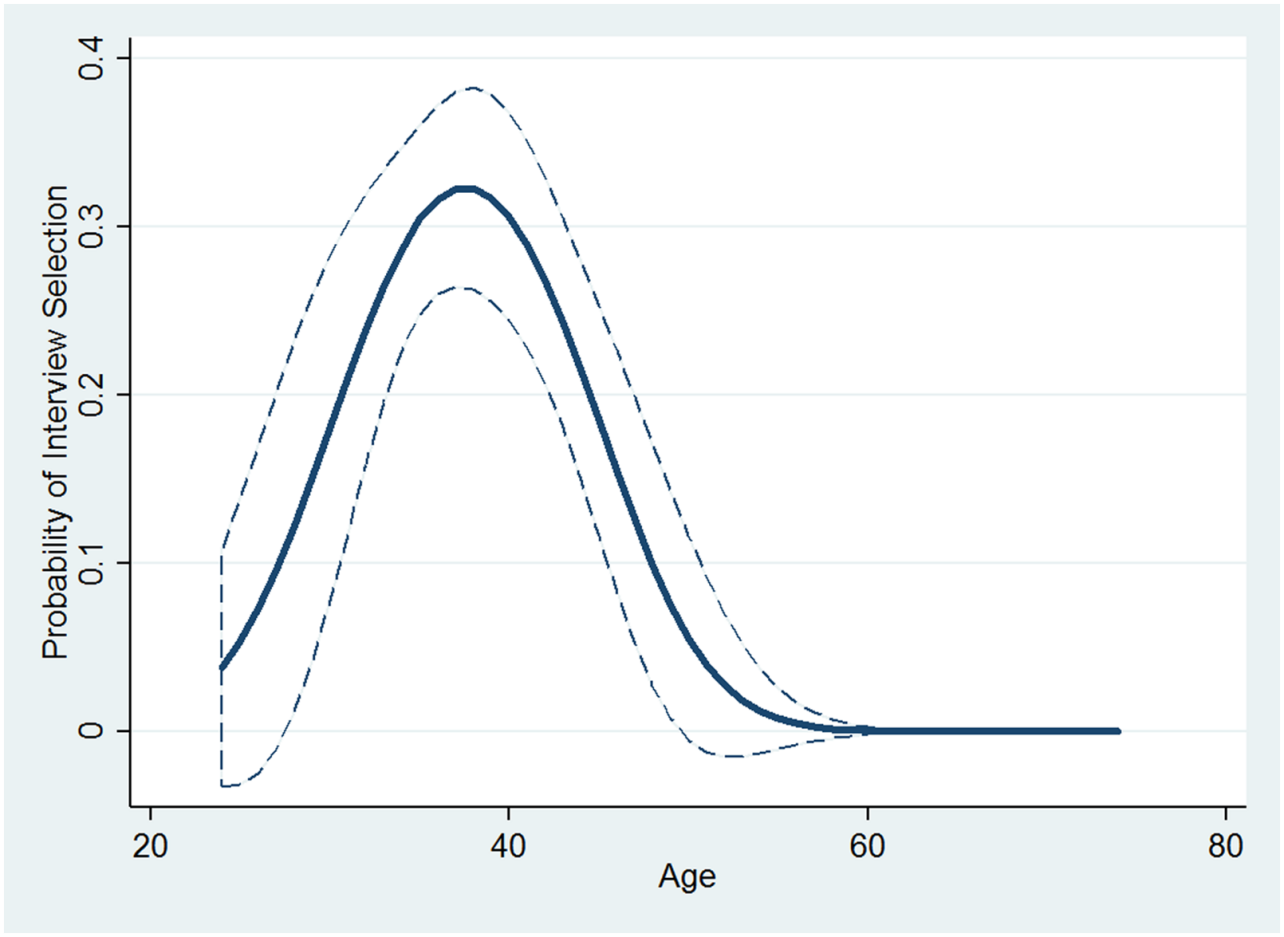
Predicted probabilities of interview selection across quadratic age, HQ only (2013 data). Dashed lines indicate 95% confidence intervals.

First, a logistic regression was used to predict whether an HQ applicant was ever interviewed (see Table 4). The results indicate that there is a nonlinear relationship between age and interview selection among HQ applicants. Fig 4 provides a useful visualization of the nonlinear relationship. The overall shape of the predicted probabilities plot is similar to the results from the 2009 data, with 40-year-old HQ applicants having the greatest likelihood of being invited for any interview and HQ applicants decreasingly likely to be invited for any interview as they increase or decrease in age relative to that.

**Table 4:** Logistic regression predicting selection for interview among HQ applicants (2013).

Variables	Base Model	Full Model
Age	2.77***	2.76***
	(0.978)	(0.979)
Age Squared	0.99***	0.99***
	(0.004)	(0.004)
Education Level		1.01
		(0.045)
Male		0.99
		(0.246)
Intercept	< 0.01***	< 0.01***
	(< 0.001)	(< 0.001)
Observations	447	447

The Base Model explores only the effects of age on interview selection. The Full Model incorporates potentially confounding covariates. Odds ratios are reported. Standard errors are in parentheses.

*** p<0.01

However, one important distinction is the absolute value of the probability of selection at the peak near 40 years of age. In the 2009 analysis, a 40-year-old applicant (including both MBQs and HQs without distinction—basically the entire pool meeting basic qualifications) had approximately a 2.5% chance of being chosen for the *second* interview (referring to Fig 1, an MBQ applicant at any age had a base probability of a first interview of ≈4.3%, a second interview of 1.7%, and HQ represented ≈17% of the MBQ pool). In the 2013 data, a 40-year-old applicant deemed HQ had approximately a 35% chance of being selected for *at least the first* interview (the base probability of an HQ applicant of any age receiving at least the first interview in 2009 was ≈25%). The qualifying statements are important because the two samples are not directly comparable, sample sizes being very different, and the outcome variable is not comparable. However, the significant increase in peak probability of interview at the age of 40 is of interest.

**Fig 5:**
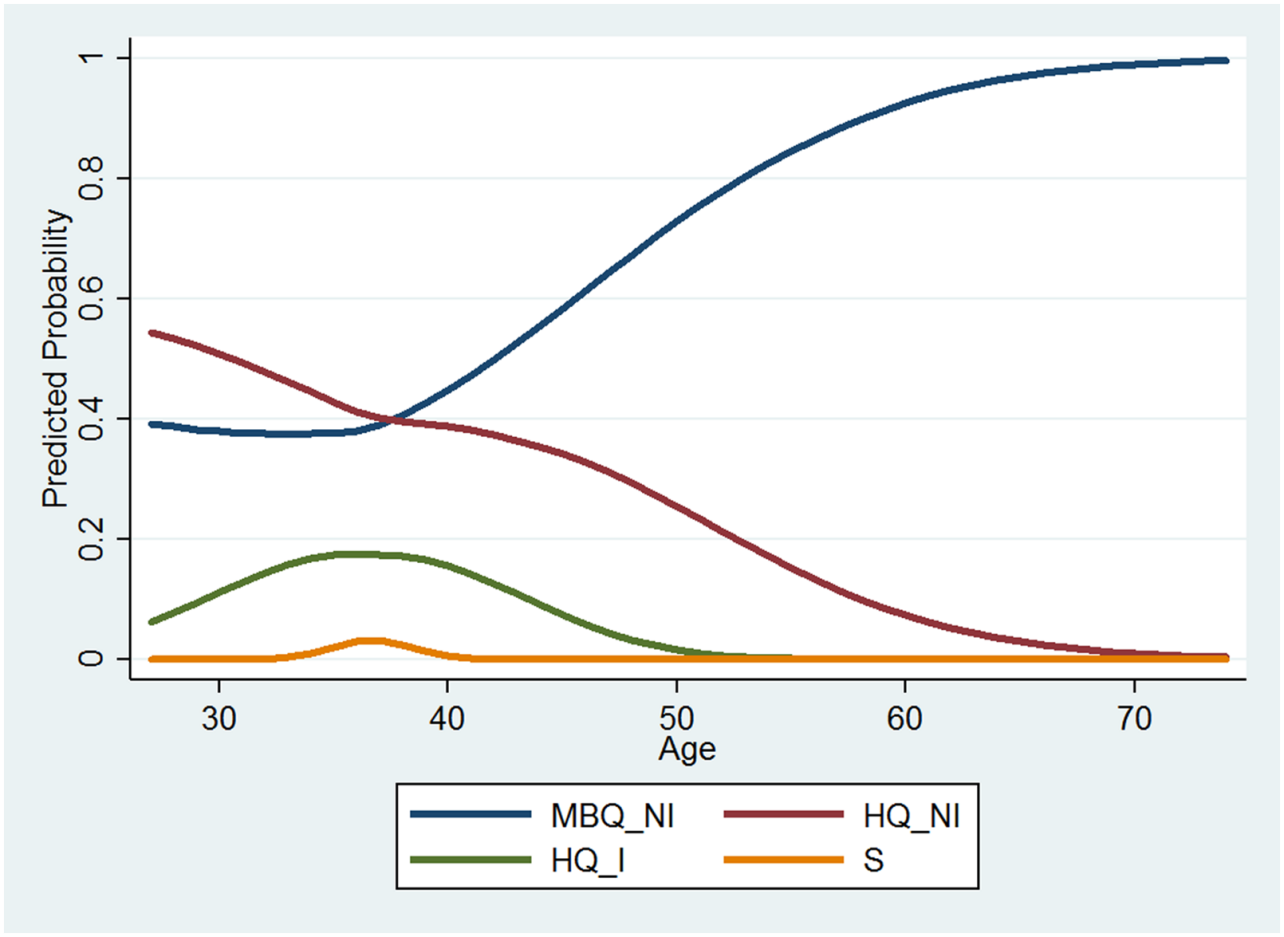
Predicted probabilities of final selection status across age across quadratic age (2013 data). No 95% confidence intervals are visible due to their small size comparable to line widths.

Tables C and D in S2 Appendix display the results of multinomial logistic regressions predicting the 4-level, categorical variable for final status of the 2013 applicants (MBQ_NI, HQ_NI, HQ_RAAFI, and S). As shown in the tables, there are both linear and nonlinear effects of age on final status revealed by the analysis. However, interpreting quadratic effects for multinomial logistic regressions can be even more complicated than for logistic regression; therefore, Fig 5 is utilized for interpretation.

Some categories of final selection status, even when including a quadratic term for age, remain linearly related to age. Specifically, the predicted probabilities for MBQ_NI (not interviewed) and HQ_NI appear linear. Conversely, there is also some evidence of nonlinear relationships. Both S and HQ_I display the familiar bump in probability just before 40 years of age with bookended declines. This curvilinear shape in the predicted probability plot suggests distinct selection processes for HQ interview selection and astronaut selection, relative to those not interviewed, regardless of qualification status.

The relationship between age and probability of not receiving an invitation for an interview appears relatively linear. As applicants age, they are less likely to obtain HQ status and not receive an interview invitation and more likely to obtain MBQ status and not receive an interview invitation. The relationship for age with interview invitation and selection appears significantly less linear. Both the probability of receiving an interview invitation and the probability of final selection into the astronaut program increase as applicants approach their late thirties, at which point the probability of receiving an interview or final selection begins to decline as they grow older beyond the age of 40. While these relationships are undoubtedly complex and difficult to interpret, they also reveal interesting dynamics in the astronaut selection process.

Another unexpected pattern found while cross-referencing applicants between the 2009 and the 2013 data was the demotion of applicants who achieved HQ status for their 2009 application, were interviewed, but subsequently lost HQ status in 2013. One might reasonably assume that applicants’ qualifications earning them an interview in 2009 would also earn them HQ status in 2013, since experience would, at a minimum, remain constant, but more likely improve over the 4 years between applicant cohorts. However, 25 applicants interviewed in 2009 were not considered HQ in 2013. As previously noted, medical disqualification information was not available and may have been a factor, although an applicant disqualified medically would not likely reapply, and furthermore, 25 of ≈120 (21%) points more toward other factors.

A chi-square analysis revealed that there was a statistically significant relationship between receiving an interview in 2009 and receiving HQ status in 2013 (see Table 5); however, the effect size of φ = 0.14 is quite small, especially for a sample size as large as 878 [27]. This result may cause concern among applicants who may have felt confident in achieving HQ status during the 2016 selection simply because they received an interview during the 2013 selection. Receiving an interview for the NASA astronaut program does not ensure that an applicant will meet HQ standards in future applications, presumably based upon interview or testing results.

**Table 5:** Crosstabulation with chi-square test for previous interview experience (2013).

	MBQ in 2013		HQ in 2013		Total	
	N	%	N	%	N	%
Not previously interviewed in 2009	406	94.2	382	85.5	788	89.8
Previously interviewed in 2009	25	5.8	65	14.5	90	10.3
Total	431	100	447	100	878	100

χ^2^ (1) = 18.2, *p* < 0.001, φ = 0.14.

### Historical data (1959–2013)

As seen in the descriptive statistics summary in Table 6, the average age at selection was 34.4 years old, the average age at retirement 48.4 years old, the average delay from selection to first flight was 6.3 years, the average time from first to last flight (operational career) was 4.4 years, and the average time from last flight to retirement was 3.6 years. The average time from selection to retirement (total astronaut career) was 14.8 years.

**Table 6:** Descriptive statistics of age milestones for NASA astronauts over all selections (1959–2013).

	N	Mean	SD	Min	Max
Age at Selection	341	34.4098	3.5898	25.2403	46.5161
Age at First Flight	337	40.8756	4.7986	32.0630	58.7817
Age at Last Flight	337	45.2969	5.3993	33.2704	61.2539
Age at Retirement	267	48.3451	7.3193	28.2382	76.6872
Years from Selection to First Flight	310	6.3018	3.0587	0.5284	19.0691
Years from First Flight to Last Flight	337	4.4213	4.2766	0.0000	18.6831
Years from First Flight to Retirement	257	8.2442	6.5000	0.0164	39.7755
Years from Selection to Last Flight	310	11.0485	4.5132	0.5284	29.2758
Years from Last Flight to Retirement	257	3.5631	4.2415	0.0164	22.4230
Years from Selection to Retirement	245	14.8070	6.9132	0.1396	42.2888

**Fig 6:**
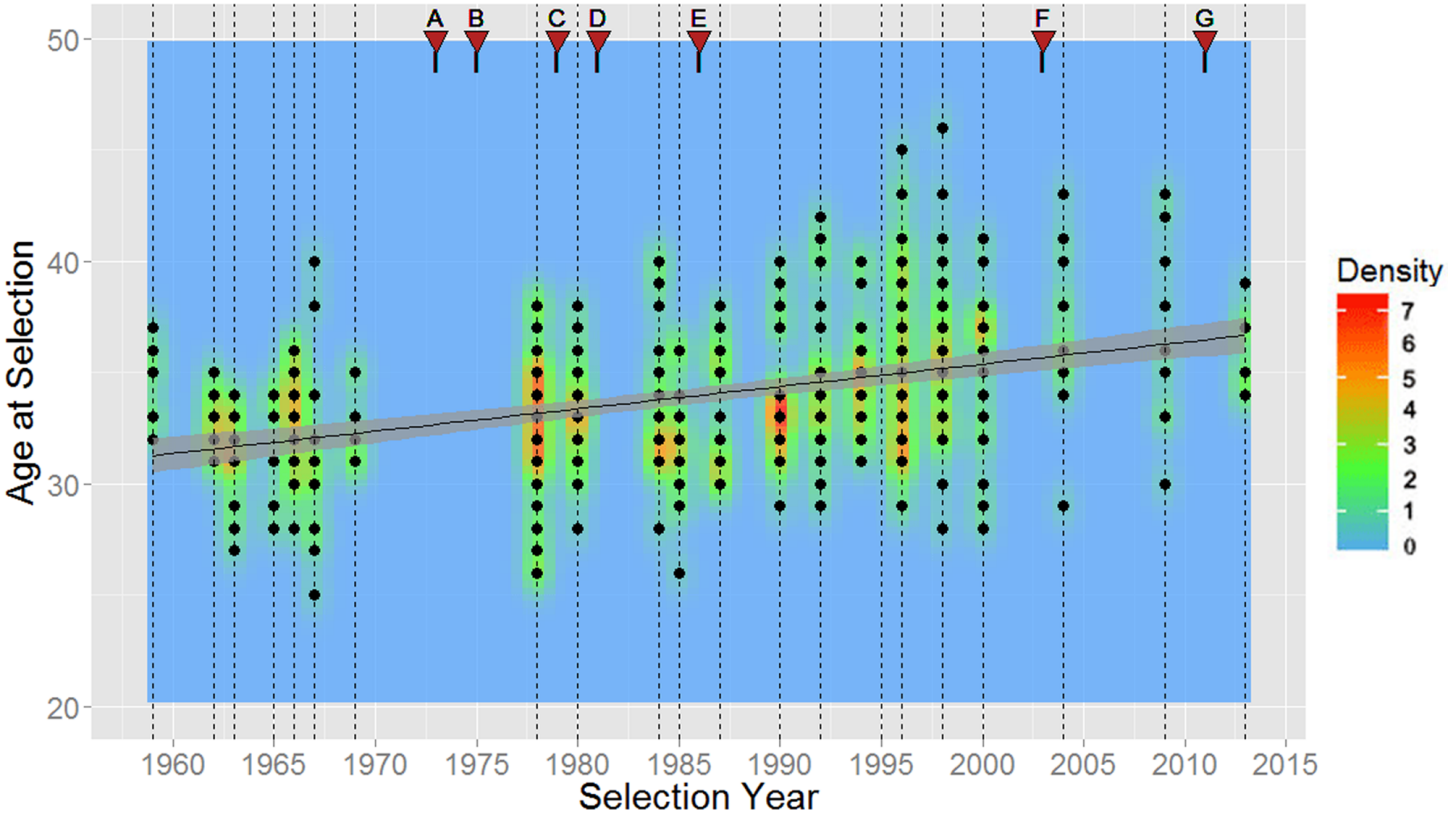
Ages of NASA astronauts at time of selection for all selections from 1959 to 2013. Dashed vertical lines represent each selection group in time, the mean age is plotted in black, and 95% confidence intervals are shaded in gray. Relevant events in NASA’s history are noted above the plot via red triangles. A: Skylab Flight Start (1973); B: Apollo Program End (1975); C: Skylab Flight Ends (1979); D: Space Shuttle Era Start (1981); E: Challenger Accident (1986); F: Columbia Accident (2003); G: Space Shuttle Era End (2011).

As shown in Table 7, NASA astronauts have been gradually getting older every selection year at their time of selection, first flight, last flight, and retirement. These relationships can also be seen in Figs 6 through 9.

**Table 7:** Pairwise Pearson’s correlations of age milestones for NASA astronauts over all selections (1959–2013).

	Age at Selection	Age at First Flight	Age at Last Flight	Age at Retirement
Selection Year	0.391***	0.222***	0.202***	0.106*
Years from Selection to First Flight	-0.026	0.631***	0.333***	0.310***
Years from First Flight to Last Flight	-0.144**	-0.296***	0.529***	0.509***
Years from First Flight to Retirement	-0.165**	-0.247***	0.393***	0.758***
Years from Selection to Last Flight	-0.154***	0.151***	0.739***	0.661***
Years from Last Flight to Retirement	-0.105	-0.081	0.057	0.647***
Years from Selection to Retirement	-0.133**	0.110*	0.557***	0.885***

*** p<0.01,

** p<0.05,

* p<0.1

**Fig 7:**
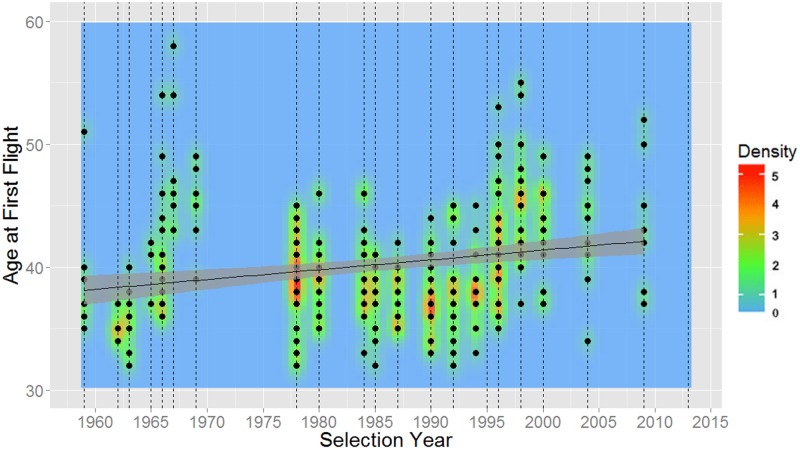
Ages of NASA astronauts at first flight versus year of selection (1959–2013). Dashed vertical lines represent each selection group in time, the mean age is plotted in black, and 95% confidence intervals are shaded in gray.

**Fig 8:**
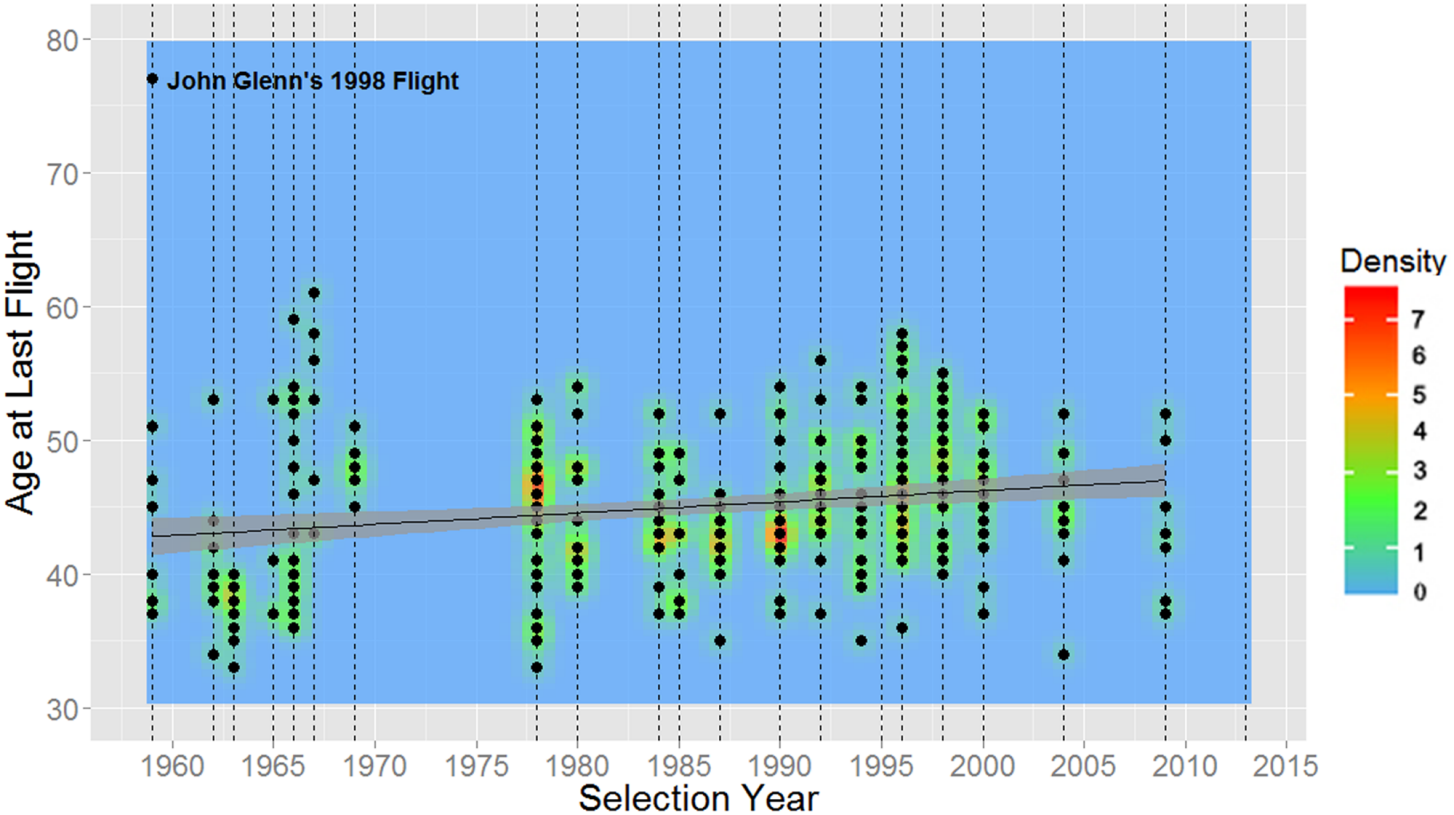
Ages of NASA astronauts at last flight versus year of selection (1959–2013). Dashed vertical lines represent each selection group in time, the mean age is plotted in black, and 95% confidence intervals are shaded in gray. A notable outlier (upper left) is Senator John Glenn, who flew on STS-95 in 1998 at the age of 77. John Glenn’s 1998 flight is included in the graph for historic accuracy, but it was not used for last flight analysis due to significant leverage on results as an extreme outlier.

**Fig 9:**
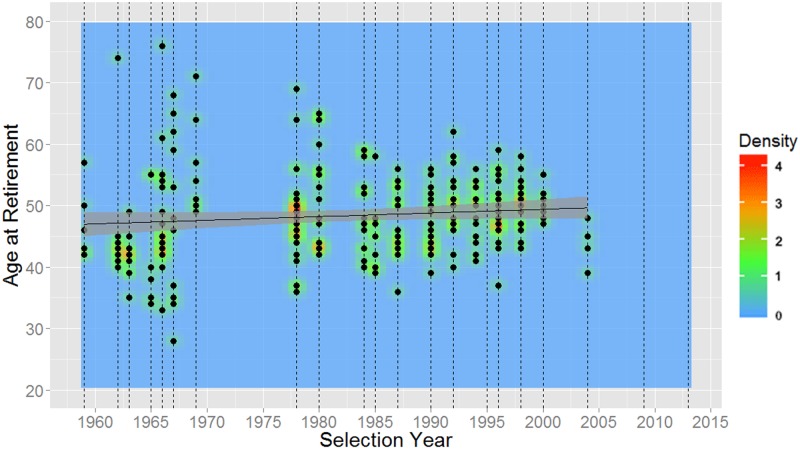
Time from selection to retirement for NASA astronauts versus year of selection (1959–2013). Dashed vertical lines represent each selection group in time, the mean age is plotted in black, and 95% confidence intervals are shaded in gray.

A number of additional relationships are also displayed in Table 7. Astronauts with more elapsed time between their selection and first flight are also older at first flight, last flight, and retirement. Astronauts with more elapsed time between their first flight and last flight, as well as astronauts with more elapsed time between their first flight and retirement, tend to be younger at their selection and first flight, but older at their last flight and retirement. Astronauts with more elapsed time between their selection and retirement, and astronauts with more elapsed time between their selection and last flight, are generally younger when selected, but older at their first flight, last flight, and retirement. Not surprisingly, astronauts with more elapsed time between their last flight and retirement are typically retire at older ages.

As a more robust test of how astronaut ages at key milestones have been trending over time, OLS regression was also performed (see Table 8). After controlling for other covariates, selection year remains positively correlated with age at selection, first flight, and last flight, though not at retirement, indicating that that astronauts have typically been getting older over time at these three milestones in their NASA careers. Males are typically older than females at these three points. Astronauts with military experience, as well as astronauts with previous NASA experience, are generally older than astronauts without military experience at their time of selection and at the time of their last flight. Having a Ph.D. has no effect on age and having an M.D. only slightly and positively affects age at selection.

**Table 8:** OLS regression of age milestones for NASA astronauts over all selections (1959–2013).

Variables	Age at Selection	Age at First Flight	Age at Last Flight	Age at Retirement
Selection Year	0.11***	0.08***	0.08***	0.02
	(0.013)	(0.022)	(0.025)	(0.040)
Male	2.57***	3.28***	2.89***	0.36
	(0.519)	(0.808)	(0.923)	(1.608)
Military Experience	1.61***	1.25	1.68*	0.02
	(0.532)	(0.825)	(0.943)	(1.565)
Previously NASA	1.43***	0.74	2.03***	1.54
	(0.440)	(0.666)	(0.761)	(1.287)
Ph.D.	0.23	0.48	1.29	0.53
	(0.490)	(0.760)	(0.869)	(1.386)
M.D.	1.02*	1.38	0.13	0.05
	(0.613)	(0.993)	(1.135)	(1.835)
Pilot	-0.58	-2.31**	1.37	6.26***
	(0.522)	(1.050)	(1.200)	(1.650)
Mission Specialist	-0.89*	-1.77*	2.00*	5.97***
	(0.477)	(0.991)	(1.134)	(1.624)
Payload Specialist	1.34*	-0.91	2.23*	3.92*
	(0.795)	(1.160)	(1.327)	(2.089)
Management	1.24**	1.01	1.43	2.30
	(0.573)	(0.849)	(0.971)	(1.979)
Constant	-192.3***	-118.1***	-125.3**	-2.6
	(26.19)	(43.56)	(49.81)	(79.54)
Observations	341	310	310	245
R-squared	0.309	0.148	0.140	0.109

Standard errors in parentheses.

*** p<0.01,

** p<0.05,

* p<0.1

The roles astronauts were assigned at NASA were also analyzed in the regressions. Pilots are typically younger than other astronauts when they take their first flight and tend to retire at an older age. Mission specialists are younger at selection and their first flight, but older at their last flight and retirement. Payload specialists, a category no longer in use, were generally older at selection, last flight, and retirement (if they remained in the program past their initial assigned flight). Astronauts in management roles are also generally older at the time of their selection, but they tend to be no older or younger than other astronauts at the other three key milestones.

## Conclusions

The analyses presented in this paper illuminate significant age bias in NASA’s astronaut selection process, whether intended or unintended. Despite a valid applicant age range typically spanning nearly five decades, selectees over many selections (1959 to 2013) have ended up within a very narrow age range. During the stepped progression from applicant pool through final selection, the most striking observation is the loss of age diversity at each stage. This bias apparently enters the process past the initial, presumably objective determination of HQ status, although even there, embedded age bias may be present (e.g., negative weighting for time elapsed after last degree).

Overall, a rather narrow age range of applicants have the highest probability of receiving an interview invitation and/or being selected into the astronaut program. Although the optimal age for maximizing an applicant’s acceptance into the astronaut program selection during the 2009 and 2013 cohorts was in the years just before their 40^th^ birthday, the future trend is somewhat uncertain. The age of those selected has been steadily climbing from 1959 through 2013, with the average age being 36.6 years in 2013. At the same time, throughout all selections over those years, the average age at retirement has been relatively steady at approximately 48 years. Together, these facts point to an increasingly narrowing operational tenure for astronauts. In addition, this suggests that one’s chances of selection diminish dramatically if he or she is already older than the average retirement age of 48.

An observation of note is that 21% (2009) and 26% (2013) of the applicant pools did not meet basic qualifications (i.e., lacked a Bachelor’s degree). In addition, some applicants who were given HQ status and granted an interview were demoted from HQ status in the subsequent application cycle. While this may simply be due to poor performance in interviews or medical disqualifications, new applicants’ HQ status is determined solely on the basis of non-interview information, suggesting a mixture of subjective and objective measures at play.

Re-interviewing of individuals, potentially on more than one application cycle, has a significant effect of reducing the chances of other applicants’ invitation to one of the few available interview spots. Thus any biases (age, race, gender, etc.) manifest via such recycling could have multiplicative effects and should be investigated.

Quite surprisingly, applicants with prior military service had an 8—10X selection advantage over those without. This may serve to guide the career paths of those interested in joining the astronaut corps, but also may elicit some discussion as to the ongoing relevance of such experience in a civilian agency.

As data becomes available, analysis should be carried out on the 2017 selection, announced while this manuscript was in review. In addition, other researchers may wish to explore the effects of gender and race on selection, alone and in combination with other factors such as age, foreign birth, and prior military experience. Regardless, it would be prudent for NASA to carry out such analyses in the interest of a fair selection processes.

While the data is not available from NASA for privacy reasons, it would also be very interesting to look at medical disqualification statistics versus age. Since the medical status of each astronaut is carefully monitored even in retirement [28], it would also be enlightening (and relatively low cost) to recall some of the many interviewees who went through the full “second interview” medical screening but were not ultimately selected, in order to illuminate potential predictive power of the screening to predict downstream disorders.

This research may provide some guidance to future applicants considering the best timing and qualifications for their application. In addition, it is suggested that NASA define and publish an upper applicant age in keeping with those of other operational federal positions. Further, NASA might also consider making more of the selection criteria, process, and anonymized statistical data public in the interest of transparency. Until then, FOIA requests seem to be the only mechanism for obtaining detailed information.

In closing, we hope that some of the methods presented herein may be productively applied to other selection processes such as those in the military and for academic admissions.

## Supporting information

S1 File FOIA data for 2009 selection obtained from NASA. (XLSX)

S2 File FOIA data for 2013 selection obtained from NASA. (XLSX)

S3 File Manually compiled astronaut demographic data across all selections. (XLSX)

S4 File R code for analyses. (RTF)

S5 File Stata code for analyses. (RTF)

S1 Appendix Additional figures. (DOCX)

S2 Appendix Additional tables. (DOCX)

S3 Appendix Glossary of variable names. (DOCX)
