# Supplementary material for: Analysis of age as a factor in NASA astronaut selection and career landmarks
Source: PLoS One. 2017 Jul 27;12(7):e0181381. doi: 10.1371/journal.pone.0181381 (PMC5531584; doi:10.1371/journal.pone.0181381)
Supplement: S4 File — (RTF) [file pone.0181381.s004.rtf]

NOTE: TO RUN THIS CODE:
Please name the data file “Astronauts Missions”. 
Replace blank spaces in the variable names with “.”
To replicate the figures exactly as published, please create rounded versions of the variables in columns I through S using the ROUNDDOWN formula in excel, rounding down to whole numbers (i.e., second parameter in ROUNDDOWN formula = 0).  

### Astronaut Plots


########################################
### heat map plots (figures 6 through 9)

# loading libraries
library(foreign)
library(ggplot2)

data<-read.csv("Astronauts Missions.csv")
data <- data[complete.cases(data$Selection.Year), ]

myColors <- colorRampPalette(c("#52a4ff", "#44ff32", "#ff9a00", "#ff0000"))


### age at Selection

breaks <- c(.002, .005, .008, .011)
labels <- c("1", "3", "5", "7")

p <- ggplot(data, aes(Selection.Year, Age.at.Selection.Round))

gm<-p + stat_density2d(geom="tile", aes(fill = ..density..), 
                       contour = FALSE, 
                       alpha = .75, h=c(2,3)) +
  geom_point(size = 3) +
  stat_smooth(method = "lm", colour = "black", alpha = .75) +
  scale_x_continuous(breaks = round(seq(min(data$Selection.Year)+1, 
                                        max(data$Selection.Year)+2, 
                                        by = 5),1)) +
  labs(title = "", x = "Selection Year", y = "Age at Selection", 
       colour="Density") + 
  geom_vline(xintercept = c(1959, 1962, 1963, 1965, 1966, 1967, 1969, 
                            1978, 1980, 1984, 1985, 1987, 1990, 1992, 
                            1995, 1996, 1998, 2000, 2004, 2009, 2013), 
             linetype = 2) +
  scale_fill_gradientn(name = "Density", colours = myColors(10), 
                       breaks=breaks, labels=labels) +
  theme(axis.text.x = element_text(size = rel(1.8)), 
        axis.text.y = element_text(size = rel(1.8)), 
        axis.title.x = element_text(size = rel(1.8)), 
        axis.title.y = element_text(size = rel(1.8), vjust=1.2), 
        legend.title=element_text(size=14)) +
  scale_y_continuous(breaks=seq(20, 50, 10), 
                     limits=c(20, 50)) +
  geom_point(x= 1973, y = 49, size = 6, shape = "I") +
  geom_point(x= 1975, y = 49, size = 6, shape = "I") +
  geom_point(x= 1979, y = 49, size = 6, shape = "I") +
  geom_point(x= 1981, y = 49, size = 6, shape = "I") +
  geom_point(x= 1986, y = 49, size = 6, shape = "I") +
  geom_point(x= 2003, y = 49, size = 6, shape = "I") +
  geom_point(x= 2011, y = 49, size = 6, shape = "I") +
  geom_point(x= 1973, y = 50, size = 5, shape = 25, fill = "firebrick") +
  geom_point(x= 1975, y = 50, size = 5, shape = 25, fill = "firebrick") +
  geom_point(x= 1979, y = 50, size = 5, shape = 25, fill = "firebrick") +
  geom_point(x= 1981, y = 50, size = 5, shape = 25, fill = "firebrick") +
  geom_point(x= 1986, y = 50, size = 5, shape = 25, fill = "firebrick") +
  geom_point(x= 2003, y = 50, size = 5, shape = 25, fill = "firebrick") +
  geom_point(x= 2011, y = 50, size = 5, shape = 25, fill = "firebrick") +
  geom_point(x= 1973, y = 51, size = 5, shape = "A") +
  geom_point(x= 1975, y = 51, size = 5, shape = "B") +
  geom_point(x= 1979, y = 51, size = 5, shape = "C") +
  geom_point(x= 1981, y = 51, size = 5, shape = "D") +
  geom_point(x= 1986, y = 51, size = 5, shape = "E") +
  geom_point(x= 2003, y = 51, size = 5, shape = "F") +
  geom_point(x= 2011, y = 51, size = 5, shape = "G") 
gm 


# Age at First Flight


breaks <- c(.0015, .006, .0105)
labels <- c("1", "3", "5")

p <- ggplot(data, aes(Selection.Year, Age.at.First.Flight.Round))

gm<-p + stat_density2d(geom="tile", aes(fill = ..density..), 
                       contour = FALSE, 
                       alpha = .75, h=c(2,3)) +
  geom_point(size = 3) +
  stat_smooth(method = "lm", colour = "black", alpha = .75) +
  scale_x_continuous(breaks = round(seq(min(data$Selection.Year)+1, 
                                        max(data$Selection.Year)+2, 
                                        by = 5),1)) + 
  labs(title = "", x = "Selection Year", y = "Age at First Flight", 
       colour="Density") + 
  geom_vline(xintercept = c(1959, 1962, 1963, 1965, 1966, 1967, 1969, 
                            1978, 1980, 1984, 1985, 1987, 1990, 1992, 
                            1995, 1996, 1998, 2000, 2004, 2009, 2013), 
             linetype = 2) +
  scale_fill_gradientn(name = "Density", colours = myColors(10), 
                       breaks=breaks, labels=labels) +
  theme(axis.text.x = element_text(size = rel(1.8)), 
        axis.text.y = element_text(size = rel(1.8)), 
        axis.title.x = element_text(size = rel(1.8)), 
        axis.title.y = element_text(size = rel(1.8), vjust=1.2), 
        legend.title=element_text(size=14)) +
  scale_y_continuous(breaks=seq(30, 60, 10), 
                     limits=c(30, 60))  
 gm 

 
# Age at Last Flight

 breaks <- c(.002, .005, .008, .011)
 labels <- c("1", "3", "5", "7")

p <- ggplot(data, aes(Selection.Year, Age.at.Last.Flight.Round))

gm<-p + stat_density2d(geom="tile", aes(fill = ..density..), 
                       contour = FALSE, 
                       alpha = .75, h=c(2,3)) +
  geom_point(size = 3) +
  stat_smooth(method = "lm", colour = "black", alpha = .75) +
  scale_x_continuous(breaks = round(seq(min(data$Selection.Year)+1, 
                                        max(data$Selection.Year)+2, 
                                        by = 5),1)) + 
  labs(title = "", x = "Selection Year", y = "Age at Last Flight", 
       colour="Density") + 
  geom_vline(xintercept = c(1959, 1962, 1963, 1965, 1966, 1967, 1969, 
                            1978, 1980, 1984, 1985, 1987, 1990, 1992, 
                            1995, 1996, 1998, 2000, 2004, 2009, 2013), 
             linetype = 2) +
  scale_fill_gradientn(name = "Density", colours = myColors(10), 
                       breaks=breaks, labels=labels) +
  theme(axis.text.x = element_text(size = rel(1.8)), 
        axis.text.y = element_text(size = rel(1.8)), 
        axis.title.x = element_text(size = rel(1.8)), 
        axis.title.y = element_text(size = rel(1.8), vjust=1.2), 
        legend.title=element_text(size=14)) +
  scale_y_continuous(breaks=seq(30, 80, 10), 
                     limits=c(30, 80))  +
  geom_point(size = 3, shape = 19, color = "black", aes(x = 1959, y = 77)) +
  annotate("text", x = 1967, y = 77, label = "John Glenn's 1998 Flight",
           fontface = 2) +
  annotate("segment", x = 1959.5, xend = 1960.5, y = 77, yend = 77,
           colour = "black")
gm


# Age at Retirement

breaks <- c(.0025, .005, .0075, .01)
labels <- c("1", "2", "3", "4")

p <- ggplot(data, aes(Selection.Year, Age.at.Retirement.Round))

gm<-p + stat_density2d(geom="tile", aes(fill = ..density..), 
                       contour = FALSE, 
                       alpha = .75, h=c(2,3)) +
  geom_point(size = 3) +
  stat_smooth(method = "lm", colour = "black", alpha = .75) +
  scale_x_continuous(breaks = round(seq(min(data$Selection.Year)+1, 
                                        max(data$Selection.Year)+2, 
                                        by = 5),1)) + 
  labs(title = "", x = "Selection Year", y = "Age at Retirement", 
       colour="Density") + 
  geom_vline(xintercept = c(1959, 1962, 1963, 1965, 1966, 1967, 1969, 
                            1978, 1980, 1984, 1985, 1987, 1990, 1992, 
                            1995, 1996, 1998, 2000, 2004, 2009, 2013), 
             linetype = 2) +
  scale_fill_gradientn(name = "Density", colours = myColors(10), 
                       breaks=breaks, labels=labels) +
  theme(axis.text.x = element_text(size = rel(1.8)), 
        axis.text.y = element_text(size = rel(1.8)), 
        axis.title.x = element_text(size = rel(1.8)), 
        axis.title.y = element_text(size = rel(1.8), vjust=1.2), 
        legend.title=element_text(size=14)) +
  scale_y_continuous(breaks=seq(20, 80, 10), 
                     limits=c(20, 80))  
gm

####################################################
### Appendix histogram plots (figures A1 through A5)

library(foreign)
library(ggplot2)

# importing 2009 foia data
data<- read.csv("Astronaut FOIA Data 2009.csv")


# figure A1

ggplot(data, aes(Age, fill = factor(Interviewed))) +
  geom_density(alpha = 0.4) + xlim(20, 75) +
  scale_fill_manual(values = c(rgb(27,158,119, maxColorValue=255), 
                               rgb(217,95,2, maxColorValue=255)), 
                    labels=c("No Final Interview\nN=2,748\nMean=37.8", "Final Interview\nN=48\nMean=38.4")) +
  theme(legend.position=c(0.88, 0.89), 
        legend.title=element_blank(), 
        legend.text = element_text(size = 15)) +  
  guides(fill = guide_legend(override.aes = list(colour = NULL))) + 
  geom_text(data = NULL, x = 25, y = .075, label = "t=0.58\np=0.564") +
  theme(axis.text.x = element_text(size = rel(1.8)), 
        axis.text.y = element_text(size = rel(1.8)), 
        axis.title.x = element_text(size = rel(1.8)), 
        axis.title.y = element_text(size = rel(1.8), vjust=1.2)) +
  labs(y = "Density")
 

### figure A2

ggplot(data, aes(Age, fill = factor(status))) +
  geom_density(alpha = 0.4) + xlim(20, 75) +
  scale_fill_manual(values = c(rgb(27,158,119, maxColorValue=255), 
                               rgb(217,95,2, maxColorValue=255), 
                               rgb(117,112,179, maxColorValue=255)), 
                    labels=c("No Final Interview\nN=2,748\nMean=37.8", "Final Interview\nN=39\nMean=38.7", "S\nN=9\nMean=37.2")) +
  theme(legend.position = c(0.88, 0.85), 
        legend.title=element_blank(), 
        legend.text = element_text(size = 15)) +  
  guides(fill = guide_legend(override.aes = list(colour = NULL))) + 
  geom_text(data = NULL, x = 25, y = .075, label = "F=0.31\np=0.735") +
  theme(axis.text.x = element_text(size = rel(1.8)), 
        axis.text.y = element_text(size = rel(1.8)), 
        axis.title.x = element_text(size = rel(1.8)), 
        axis.title.y = element_text(size = rel(1.8), vjust=1.2)) +
  labs(y = "Density")


### importing 2013 foia data
data<- read.csv("Astronaut FOIA Data 2013.csv")

data_na<-data[order(data$Interviewed),]
data_na<-data_na[432:878,]


### figure A3

ggplot(data_na, aes(Age, fill = factor(Interviewed))) +
  geom_density(alpha = 0.4) + xlim(20, 75) +
  scale_fill_manual(values = c(rgb(27,158,119, maxColorValue=255), 
                               rgb(217,95,2, maxColorValue=255), 
                               rgb(217,95,2, maxColorValue=255)), 
                    labels=c("HQ_NI\nN=332\nMean=39.0", 
                             "HQ_I\nN=115\nMean=37.8")) +
  theme(legend.position=c(0.91,0.9), 
        legend.title=element_blank(), 
        legend.text = element_text(size = 15)) +  
  guides(fill = guide_legend(override.aes = list(colour = NULL))) + 
  geom_text(data = NULL, x = 25, y = .075, label = "t=2.10\np=0.036") +
  theme(axis.text.x = element_text(size = rel(1.8)), 
        axis.text.y = element_text(size = rel(1.8)), 
        axis.title.x = element_text(size = rel(1.8)), 
        axis.title.y = element_text(size = rel(1.8), vjust=1.2)) +
  labs(y = "Density")


### figure A4

ggplot(data, aes(Age, fill = factor(status))) +
  geom_density(alpha = 0.4) + xlim(20, 75) +
  scale_fill_manual(values = c(rgb(27,158,119, maxColorValue=255), 
                               rgb(217,95,2, maxColorValue=255), 
                               rgb(117,112,179, maxColorValue=255), 
                               rgb(228,26,28, maxColorValue=255)), 
                    labels=c("MBQ_NI\nN=431\nMean=42.0",
                             "HQ_NI\nN=332\nMean=39.0", 
                             "HQ_RAAFI\nN=107\nMean=37.9", 
                             "S\nN=8\nMean=36.6")) +
  theme(legend.position=c(0.92,0.8), 
        legend.title=element_blank(), 
        legend.text = element_text(size = 15)) +  
  guides(fill = guide_legend(override.aes = list(colour = NULL))) + 
  geom_text(data = NULL, x = 25, y = .075, label = "F=19.16\np<0.001") +
  theme(axis.text.x = element_text(size = rel(1.8)), 
        axis.text.y = element_text(size = rel(1.8)), 
        axis.title.x = element_text(size = rel(1.8)), 
        axis.title.y = element_text(size = rel(1.8), vjust=1.2)) +
  labs(y = "Density")
