# Supplementary material for: Analysis of age as a factor in NASA astronaut selection and career landmarks
Source: PLoS One. 2017 Jul 27;12(7):e0181381. doi: 10.1371/journal.pone.0181381 (PMC5531584; doi:10.1371/journal.pone.0181381)
Supplement: S5 File — (RTF) [file pone.0181381.s005.rtf]

*************************
*** creating the random sample variable
*************************

set seed 8675309
gen rando = runiform()
sort status rando
gen insamp = _n <= 400
replace insamp = 1 if status > 1


*********************
*** 2009 data preliminary analysis
*********************

* t test
ttest Age, by(Interviewed)

* anova
oneway Age Status, tabulate
pwmean Age, over(Status) mcompare(tukey) effects

*********************
*** 2009 data primary analysis
*********************

* interview logit analysis
logit Interviewed c.Age, or
logit Interviewed c.Age c.Education i.NASA i.Military i.Gender, or
logit Interviewed c.Age if insamp == 1, or
logit Interviewed c.Age c.Education i.NASA i.Military i.Gender if insamp == 1, or

* interview logit analysis with age quadradic
logit Interviewed c.Age##c.Age, or
logit Interviewed c.Age##c.Age c.Education i.NASA i.Military i.Gender, or
logit Interviewed c.Age##c.Age if insamp == 1, or
logit Interviewed c.Age##c.Age c.Education i.NASA i.Military i.Gender if insamp == 1, or

* Status multinomial logit analysis
mlogit Status c.Age, rrr
mlogit Status c.Age c.Education i.NASA i.Military i.Gender, rrr
mlogit Status c.Age if insamp == 1, rrr
mlogit Status c.Age c.Education i.NASA i.Military i.Gender if insamp == 1, rrr

* Status multinomial logit analysis with age quadratic
mlogit Status c.Age##c.Age, rrr
mlogit Status c.Age##c.Age c.Education i.NASA i.Military i.Gender, rrr
mlogit Status c.Age##c.Age if insamp == 1, rrr
mlogit Status c.Age##c.Age c.Education i.NASA i.Military i.Gender if insamp == 1, rrr

* Status multinomial logit analysis with age quadratic, changing the base category
mlogit Status c.Age##c.Age, base(2) rrr
mlogit Status c.Age##c.Age c.Education i.NASA i.Military i.Gender, base(2) rrr
mlogit Status c.Age##c.Age if insamp == 1, base(2) rrr
mlogit Status c.Age##c.Age c.Education i.NASA i.Military i.Gender if insamp == 1, base(2) rrr


*** creating the graphs

logit Interviewed c.Age##c.Age c.Education i.NASA i.Military i.Gender, or
margins , at(Age=(24(1)74)) vsquish
marginsplot, recast(line) recastci(rarea) 

mlogit Status c.Age##c.Age c.Education i.NASA i.Military i.Gender if insamp == 1, rrr
margins , at(Age=(24(1)74)) vsquish
marginsplot, recast(line) recastci(rarea) 


******************************** 
*** Sensitivity Analyses 2009
********************************

firthlogit Interviewed Age, or
firthlogit Interviewed Age Age2 Education NASA Military Gender, or
firthlogit Interviewed Age if insamp == 1, or
firthlogit Interviewed Age Age2 Education NASA Military Gender if insamp == 1, or

**********************************
*** 2013 data preliminary analysis
**********************************

* t test
ttest Age, by(Interviewed)

* anova
oneway Age Status, tabulate
pwmean Age, over(Status) mcompare(tukey) effects

******************************
*** 2013 data primary analysis
******************************

* interview
logit Interviewed c.Age##c.Age, or
logit Interviewed c.Age##c.Age c.Education i.Gender, or

* Status 
mlogit Status c.Age c.Education i.Gender, rrr
mlogit Status c.Age##c.Age c.Education i.Gender, rrr

* crosstab previous interview
tab PreviousInterview HQ, chi col 

*** creating the graphs

logit Interviewed c.Age##c.Age c.Education i.Gender, or
margins , at(Age=(24(1)74)) vsquish
marginsplot, recast(line) recastci(rarea) 

mlogit Status c.Age##c.Age c.Education i.Gender, rrr
margins , at(Age=(27(1)74)) vsquish
marginsplot, recast(line) recastci(rarea)

mlogit Status c.Age c.Education i.Gender, rrr
margins , at(Age=(27(1)74)) 
marginsplot, recast(line) recastci(rarea)


********************************
*** Age over all groups analyses
********************************

sum AgeatSelection AgeatRetirement AgeatFirstFlight AgeatLastFlight ///
SelectionAnnouncementDatetoF FirstFlightLaunchDatetoLast FirstFlightLaunchDatetoReti ///
SelectionAnnouncementDatetoR SelectionAnnouncementDatetoL ///
LastFlightLaunchDatetoRetir

pwcorr AgeatSelection AgeatRetirement AgeatFirstFlight AgeatLastFlight ///
SelectionAnnouncementDatetoF FirstFlightLaunchDatetoLast FirstFlightLaunchDatetoReti ///
SelectionAnnouncementDatetoR SelectionAnnouncementDatetoL ///
LastFlightLaunchDatetoRetir, sig

encode Gender, gen(gender)
replace Announcement = "" if Announcement == "Not Announced"

generate andate = date(Announcement, "MDY")
format %td andate

gen anyr=year(andate)
gen anyr=year(Announcement)

reg AgeatSelection anyr gender Military PreviousNASAExperienceBefore PhD MD ///
NASAPilotFromCrewLog MissionSpecialistFromCrew PayloadSpecialistFromCrewL ///
NASAManagementFromBio 
reg AgeatFirstFlight anyr gender Military PreviousNASAExperienceBefore PhD MD ///
NASAPilotFromCrewLog MissionSpecialistFromCrew PayloadSpecialistFromCrewL ///
NASAManagementFromBio 
reg AgeatLastFlight anyr gender Military PreviousNASAExperienceBefore PhD MD ///
NASAPilotFromCrewLog MissionSpecialistFromCrew PayloadSpecialistFromCrewL ///
NASAManagementFromBio 
reg AgeatRetirement anyr gender Military PreviousNASAExperienceBefore PhD MD ///
NASAPilotFromCrewLog MissionSpecialistFromCrew PayloadSpecialistFromCrewL ///
NASAManagementFromBio
