# Supplementary material for: Analysis of age as a factor in NASA astronaut selection and career landmarks
Source: PLoS One. 2017 Jul 27;12(7):e0181381. doi: 10.1371/journal.pone.0181381 (PMC5531584; doi:10.1371/journal.pone.0181381)
Supplement: S1 Appendix — (DOCX) [file pone.0181381.s006.docx]

**S1 Appendix**


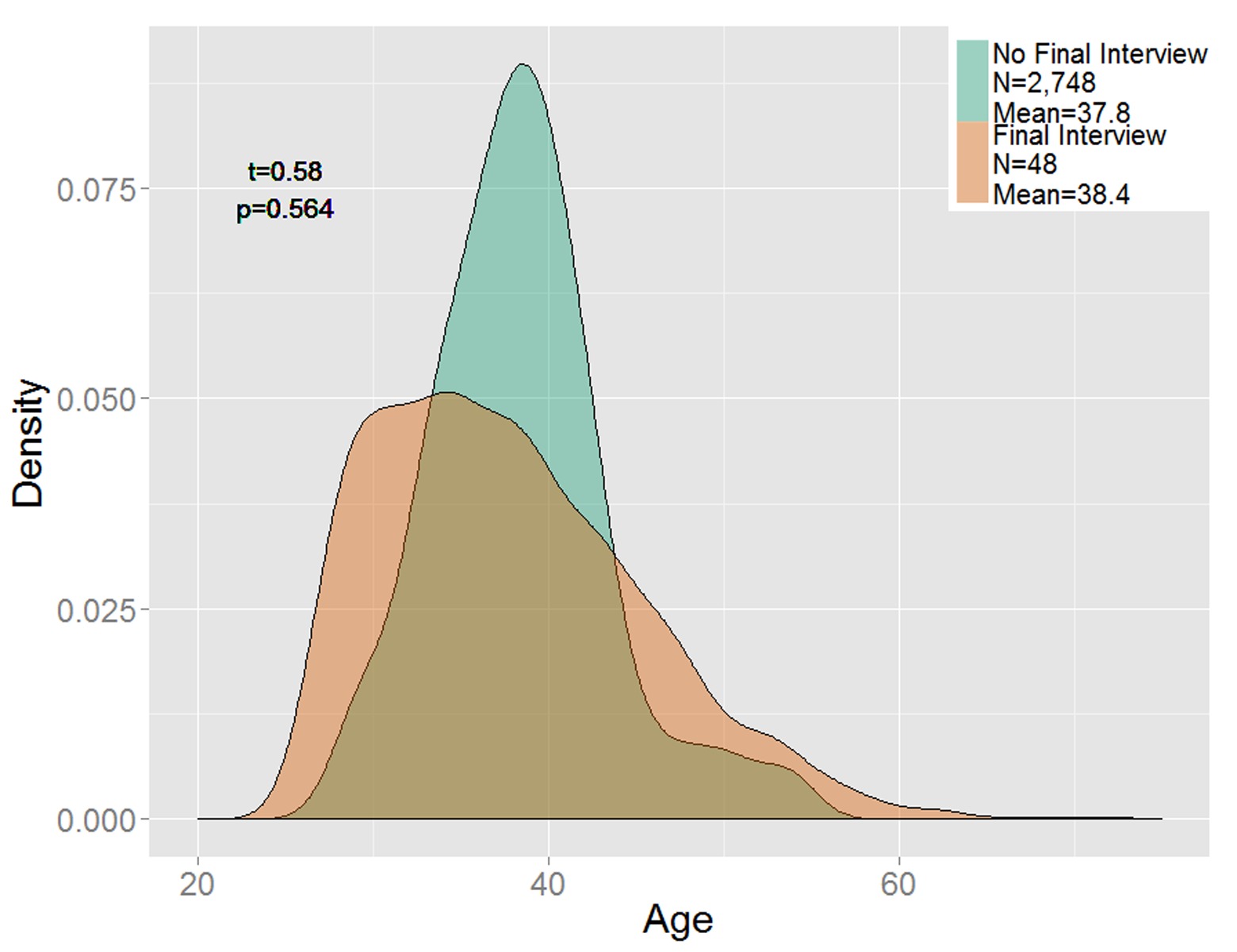


**Fig A. Independent samples t-test comparing age across final interview status (2009 data).**

**
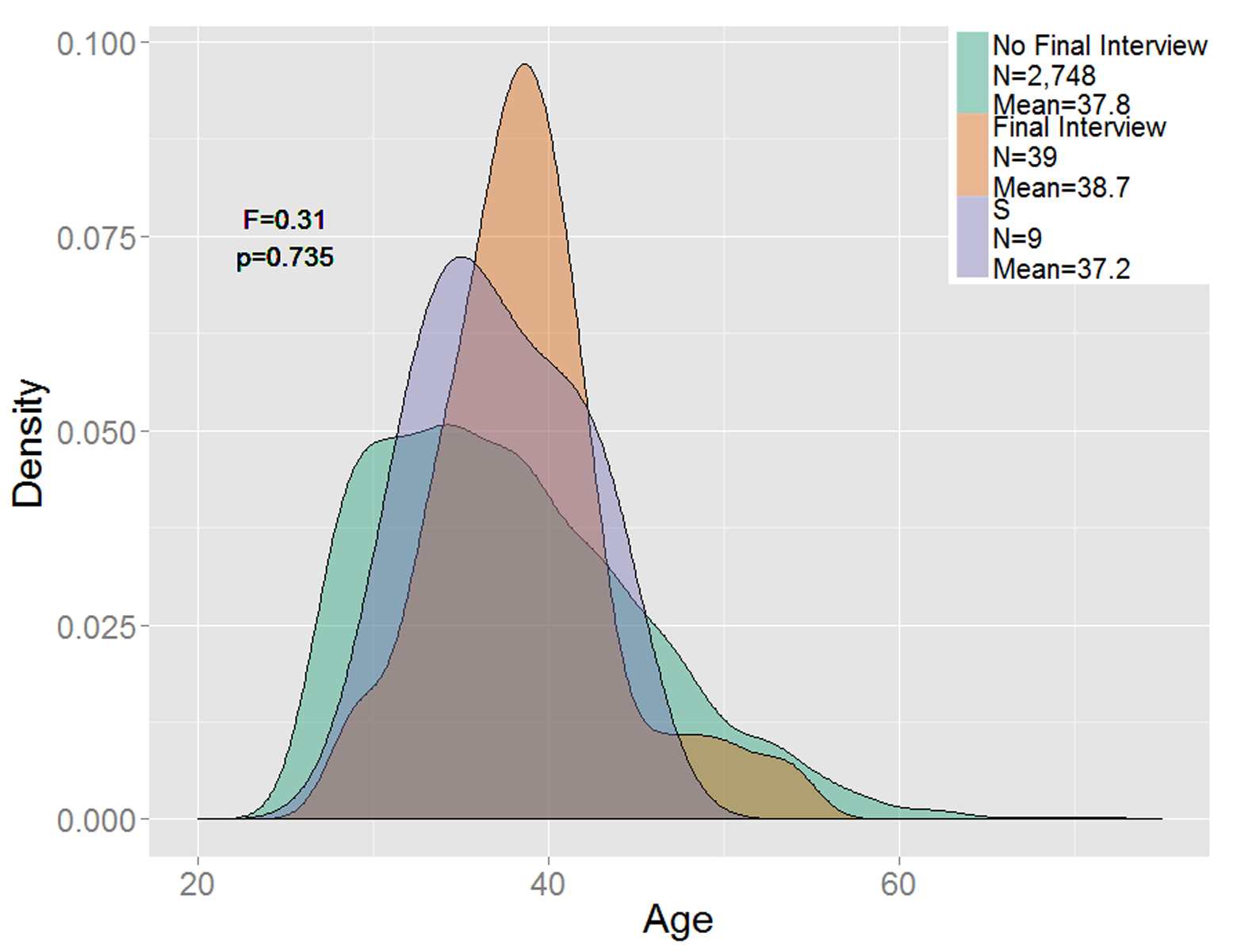
**

**Fig B. One-way ANOVA comparing age across selection stages (2009 data).**

**
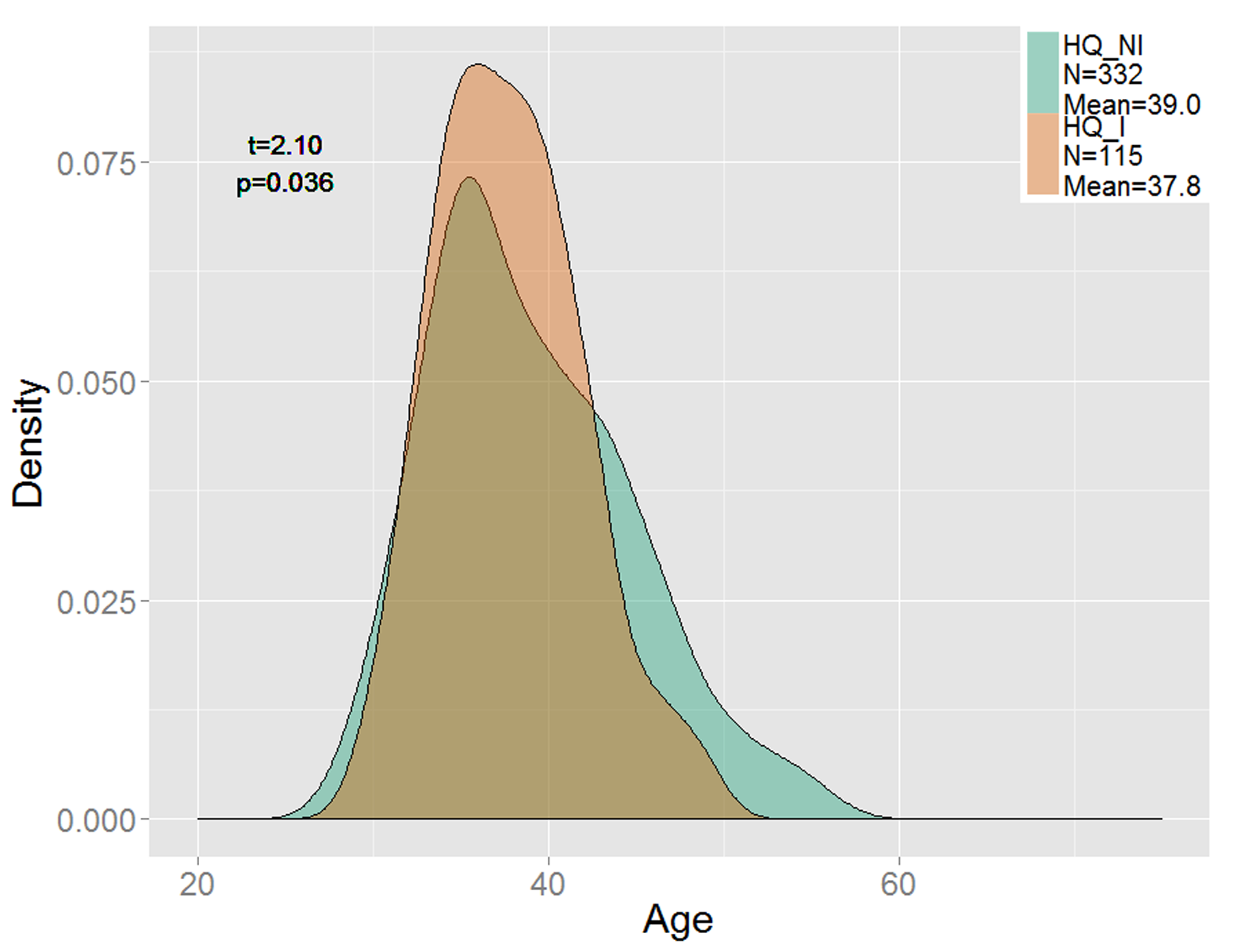
**

**Fig C. Independent samples t-test comparing age across final interview status (2013 data).**

**
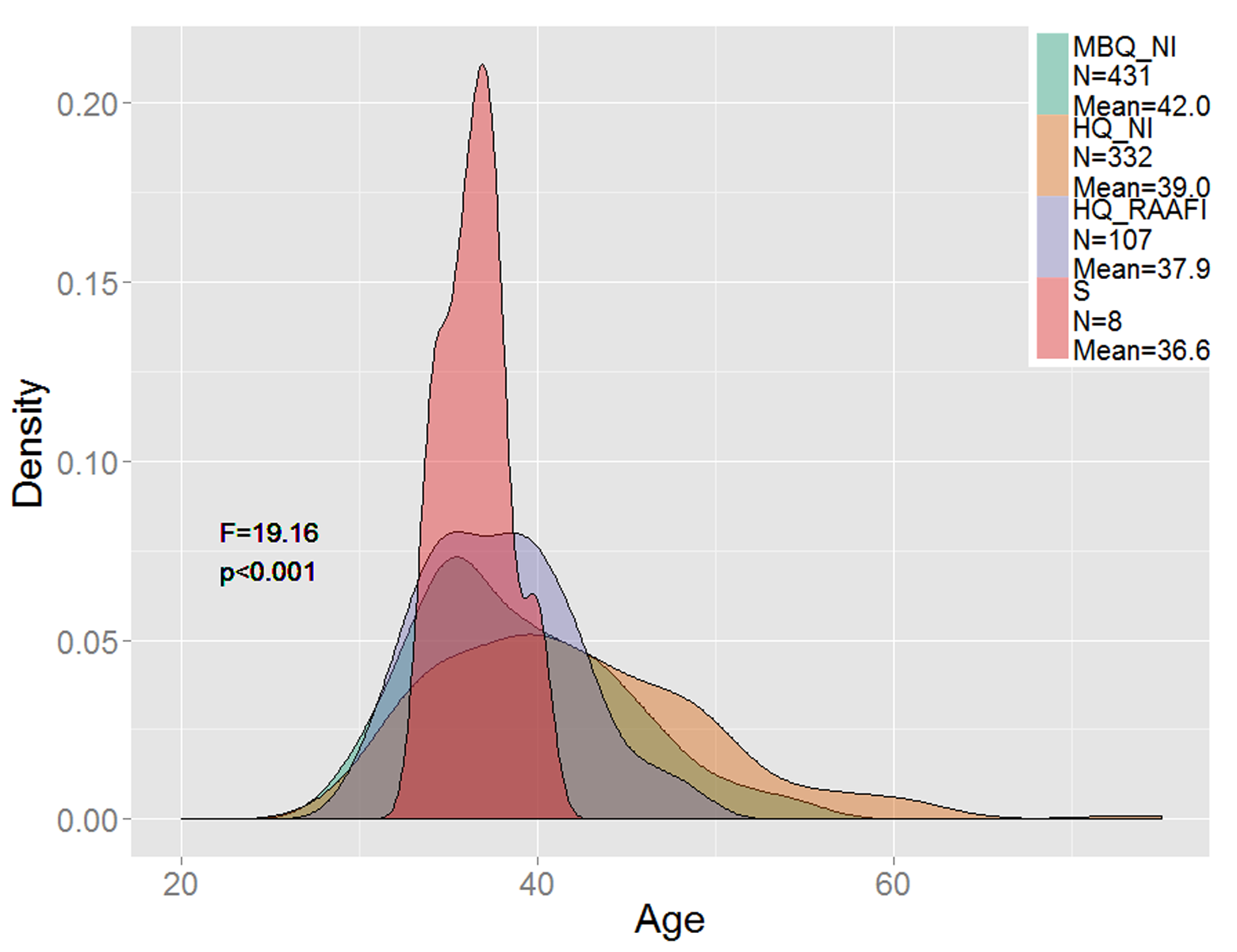
**

**Fig D. One-way ANOVA comparing age across selection stages (2013 data).**

**
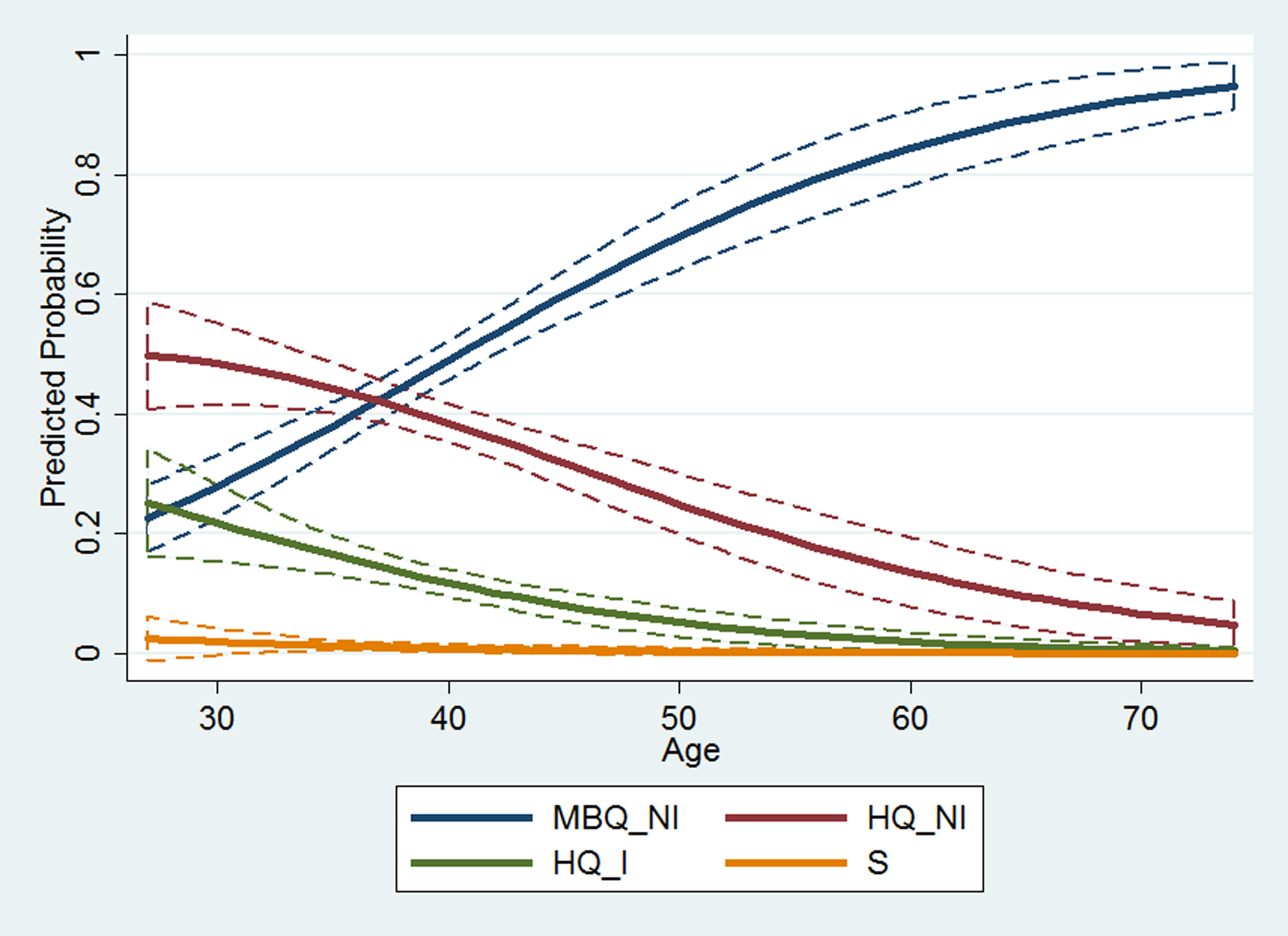
**

**Fig E. Predicted probabilities of final selection status across age (2013 data).**

Dashed lines indicate 95% confidence intervals.
