# Supplementary material for: Analysis of age as a factor in NASA astronaut selection and career landmarks
Source: PLoS One. 2017 Jul 27;12(7):e0181381. doi: 10.1371/journal.pone.0181381 (PMC5531584; doi:10.1371/journal.pone.0181381)
Supplement: S2 Appendix — (DOCX) [file pone.0181381.s007.docx]

**S2 Appendix**

**Table A. Logistic Regression Predicting Selection for Final Interview (2009**)

|  | **Full Sample** | | **Random Sample** | |
| --- | --- | --- | --- | --- |
|  | **Base Model** | **Full Model** | **Base Model** | **Full Model** |
| Age | 1.01 | 1.01 | 1.01 | 1.01 |
|  | (0.019) | (0.024) | (0.020) | (0.025) |
| Education Level |  | 1.15*** |  | 1.19*** |
|  |  | (0.051) |  | (0.062) |
| NASA Experience |  | 2.39* |  | 2.84** |
|  |  | (1.117) |  | (1.459) |
| Military Service |  | 10.43*** |  | 11.71*** |
|  |  | (3.475) |  | (4.584) |
| Male |  | 0.73 |  | 0.93 |
|  |  | (0.284) |  | (0.388) |
| Intercept | 0.01*** | < 0.01*** | 0.10*** | 0.01*** |
|  | (0.008) | (0.003) | (0.074) | (0.014) |
| Observations | 2,796 | 2,796 | 448 | 448 |

Note: Base Models explore only the effect of age on selection for final interview. Full Models incorporate potentially confounding covariates. Odds ratios are reported. Standard errors are in parentheses. *** p<0.01, ** p<0.05, * p<0.1

**Table B. Multinomial Logistic Regression Predicting Final Selection Status (2009)**

|  | **Full Sample** | | **Random Sample** | |
| --- | --- | --- | --- | --- |
|  | **Base Model** | **Full Model** | **Base Model** | **Full Model** |
| **Rejected at Final Interview** | | | | |
| Age | 1.02 | 1.01 | 1.01 | 1.01 |
|  | (0.021) | (0.026) | (0.022) | (0.027) |
| Education Level |  | 1.14*** |  | 1.18*** |
|  |  | (0.055) |  | (0.066) |
| NASA Experience |  | 2.95** |  | 3.44** |
|  |  | (1.409) |  | (1.790) |
| Military Service |  | 9.06*** |  | 10.06*** |
|  |  | (3.366) |  | (4.268) |
| Male |  | 0.97 |  | 1.22 |
|  |  | (0.450) |  | (0.595) |
| Intercept | 0.01*** | < 0.01*** | 0.06*** | 0.01*** |
|  | (0.006) | (0.002) | (0.055) | (0.010) |
| **Selected into Astronaut Program** | | | | |
| Age | 0.99 | 0.98 | 0.98 | 0.99 |
|  | (0.045) | (0.059) | (0.046) | (0.061) |
| Education Level |  | 1.22* |  | 1.29** |
|  |  | (0.127) |  | (0.151) |
| NASA Experience |  | < 0.01 |  | < 0.01 |
|  |  | (0.004) |  | (0.002) |
| Military Service |  | 19.77*** |  | 25.13*** |
|  |  | (14.680) |  | (21.620) |
| Male |  | 0.25* |  | 0.32 |
|  |  | (0.194) |  | (0.256) |
| Intercept | <0.01*** | <0.01*** | 0.04* | <0.01** |
|  | (0.008) | (0.003) | (0.073) | (0.011) |
| Observations | 2,796 | 2,796 | 448 | 448 |

Note: Base Models explore only the effects of age on final selection status. Full Models incorporate potentially confounding covariates. MBQ and HQ but not invited for the final interview is the reference category. Relative risk ratios are reported. Standard errors are in parentheses. *** p<0.01, ** p<0.05, * p<0.1

**Table C. Multinomial Logistic Regression Predicting Final Status (2013)**

|  | **HQ, not Invited for an Interview** | **HQ, Rejected After Interview** | **HQ, Selected as Astronaut** |
| --- | --- | --- | --- |
| Age | 0.92*** | 0.88*** | 0.86** |
|  | (0.012) | (0.019) | (0.064) |
| Education Level | 1.22*** | 1.25*** | 1.19 |
|  | (0.037) | (0.053) | (0.170) |
| Male | 0.65** | 0.71 | 0.26* |
|  | (0.121) | (0.187) | (0.192) |
| Intercept | 6.80*** | 7.74** | 4.72 |
|  | (3.554) | (6.166) | (12.678) |
| Observations | 332 | 107 | 8 |

Note: MBQ only (*N* = 431) is the reference category for the dependent variable. Relative risk ratios are reported. Standard errors are in parentheses. *** p<0.01, ** p<0.05, * p<0.1

**Table D. Multinomial Logistic Regression Predicting Final Status Including Quadratic Term for Age (2013)**

|  | **HQ, not Invited for an Interview** | **HQ, Rejected After Interview** | **HQ, Selected as Astronaut** |
| --- | --- | --- | --- |
| Age | 1.15 | 2.89*** | 91,020.66* |
|  | (0.156) | (0.987) | (565,660.300) |
| Age^2^ | 1.00* | 0.99*** | 0.86* |
|  | (0.002) | (0.004) | (0.072) |
| Education Level | 1.21*** | 1.24*** | 1.18 |
|  | (0.037) | (0.054) | (0.169) |
| Male | 0.63** | 0.67 | 0.23** |
|  | (0.118) | (0.177) | (0.168) |
| Intercept | 0.06 | < 0.01*** | < 0.01* |
|  | (0.174) | (< 0.001) | (< 0.001) |
| Observations | 332 | 107 | 8 |

Note: MBQ only (*N* = 431) is the reference category for the dependent variable. Relative risk ratios are reported. Standard errors are in parentheses. *** p<0.01, ** p<0.05, * p<0.1
