# Supplementary material for: Analysis of age as a factor in NASA astronaut selection and career landmarks
Source: PLoS One. 2017 Jul 27;12(7):e0181381. doi: 10.1371/journal.pone.0181381 (PMC5531584; doi:10.1371/journal.pone.0181381)
Supplement: S3 Appendix — (DOCX) [file pone.0181381.s008.docx]

**S3 Appendix**

General Variable Acronyms

MBQ: Meets Basic Qualifications

HQ: Highly Qualified

HQ_I: Highly Qualified and Granted an Interview

HQ_IR: Highly Qualified, Granted an Interview, Rejected

2009-Specific Variable Acronyms

S: Astronaut Program Selectee

HQ_SI: Highly Qualified and Granted a Second Interview

ALMBQ_NSI: At Least Met Basic Qualifications but Not Granted a Second Interview

2013-Specific Variable Acronyms

S: Astronaut Program Selectee

MBQ_NI: Met Basic Qualifications but Not Interviewed

HQ_NI: Highly Qualified but Not Interviewed

HQ_RAAFI: Highly Qualified but Rejected After At Least First Interview
